# Integrating multi-omics with regular analyses to elucidate the effects of ZnO nanomaterials on improving soybean quality

**DOI:** 10.1186/s40643-026-01109-1

**Published:** 2026-08-05

**Authors:** Xinqi Hu, Yi Zhao, Shixin Cai, Yuting Zhang, Xiaoli Wang, Nandi Zhou

**Affiliations:** School of Biotechnology and Key Laboratory of Carbohydrate Chemistry and Biotechnology of Ministry of Education, Jiangnan University, Wuxi, 214122 China

**Keywords:** Zinc oxide nanomaterials, Soybean quality, Multi-omics, Isoflavones

## Abstract

With rising global demand for high-quality food crops, soybean production is constrained by an inherent yield-quality trade-off that conventional practices cannot easily overcome. Zinc oxide nanomaterials (ZnO NMs) show great potential for crop improvement, but their molecular mechanism underlying soybean quality regulation remains unclear. This study investigated the effects of ZnO quantum dots (ZnO QDs, 5.56 nm) and ZnO nanoparticles (ZnO NPs, 29.68 nm) on soybean growth and quality via root and foliar application with different concentrations (0, 5, 10, 20, 50 and 100 mg/kg). Among these experimental groups, root application of 50 mg/kg ZnO QDs (R-QD-50) was selected as the optimal combination based on regular analyses and used for integrated transcriptomic, proteomic and metabolomic analyses. The results showed that ZnO NMs significantly promoted soybean growth, yield and quality in ZnO NMs type, application mode and concentration-dependent manner. Multi-omics integration identified three common pathways: taurine and hypotaurine metabolism, butanoate metabolism, and phenylpropanoid biosynthesis. Among them, only phenylpropanoid biosynthesis formed a complete transcriptome-proteome-metabolome regulatory chain. Key genes *phenylalanine ammonia lyase* (*PAL*) and *cinnamic acid-4-hydroxylase* (*C4H*) were significantly upregulated, the related synthases increased by ~ 35% at the protein level, and downstream metabolites including isoflavones, lignin and phenolic acids were increased by ~ 52%. Overall, this study clarified the potential molecular mechanism of ZnO NMs in improving soybean quality and provided theoretical reference for the application of ZnO NMs in soybean quality improvement under controlled pot conditions.

**Graphical abstract:**

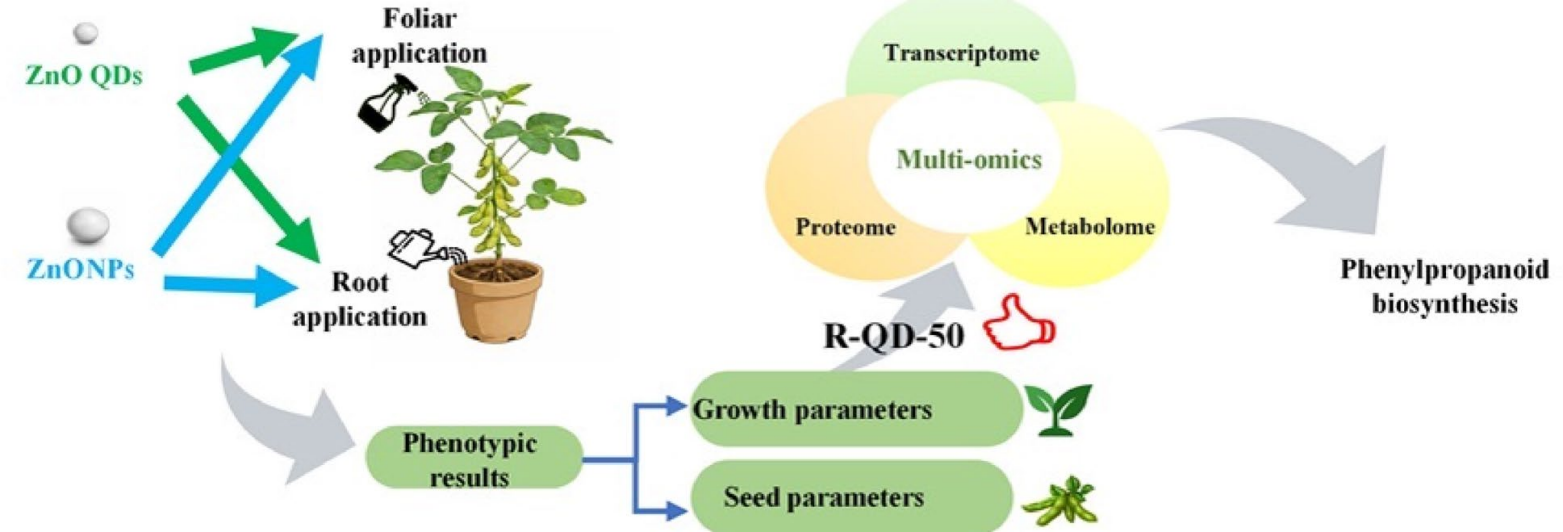

**Supplementary Information:**

The online version contains supplementary material available at 10.1186/s40643-026-01109-1.

## Introduction

Global food supply faces challenges with the growing demand for high-quality nutritious crops. As a key plant protein source, soybean (*Glycine max*) is vital for human nutrition and animal feed, but traditional breeding and cultivation struggle to meet quality demands (Sun et al. 2025). The development of soybean multi-omics resources has laid a foundation for exploring innovative quality improvement strategies and elucidating molecular mechanisms (Liu et al. 2023).

Nanotechnology has emerged as a revolutionary and sustainable approach in modern agriculture, offering novel solutions to address the challenges of low crop productivity, nutrient inefficiency, and environmental stressors (do Couto Junior et al. 2025). Nanomaterials with their unique nanoscale properties, including small size, large specific surface area, and high bioactivity, are widely applied in agricultural production, mainly serving as nanofertilizers, nanopesticides, nanocarriers, and plant growth regulators (Elsabagh et al. 2024; Wen et al. 2025). Their core regulatory mechanisms in crops include enhancing nutrient uptake efficiency, modulating gene expression and physiological metabolism, and activating antioxidant defense systems to alleviate abiotic stress, thereby promoting plant growth, yield and nutrient accumulation (Francis et al. 2024).

Among the various nanomaterials used in agriculture, zinc oxide nanomaterials (ZnO NMs) are particularly promising due to their dual role as nanocarriers and bioavailable zinc supplements (Guo et al. 2024). Compared with conventional zinc fertilizers, ZnO NMs exhibit superior efficacy in regulating plant growth, yield and stress resistance (Upadhyay et al. 2023).

Accumulating evidence confirms that ZnO NMs also improve nutritional quality across diverse crops, such as increased protein and starch content in cereals, elevated antioxidant levels in vegetables, and enhanced flavor in fruits (Adrees et al. 2021; Ouzakar et al. 2024; Zafar et al. 2022). These effects are underpinned by the essential role of zinc in protein synthesis and carbohydrate metabolism, which highlights the potential of ZnO NMs for soybean quality improvement (Cheng et al. 2025; Raza et al. 2025). The efficacy of ZnO NMs can be further governed by their inherent properties, application modes, and concentrations. For instance, different particle sizes confer distinct functions: ZnO quantum dots (ZnO QDs) have high bioavailability, while larger ZnO nanoparticles (ZnO NPs) show better soil stability (Sembada & Lenggoro 2024; Wang et al. 2022). Additionally, application modes (root *vs.* foliar) also influence plant absorption efficiency and physiological regulation (Bian et al. 2026).

In recent years, multi-omics strategies (including transcriptomics, proteomics and metabolomics) have emerged as powerful tools to decode plant-environment interactions, with proven efficacy in systematically resolving the transcriptional and metabolic regulatory networks of nutrient and functional metabolite biosynthesis in crops (Han et al., 2025; Li et al. 2025). These strategies have also been widely applied to dissect nanomaterial-crop interactions, achieving remarkable progress in elucidating how nanomaterials regulate crop growth, stress resilience and nutritional quality (Sun et al. 2020).Transcriptomic and metabolomic analyses have revealed that ZnO NMs modulate plant growth in tomato, whereas selenium nanoparticles (Se NPs) improve the nutritional quality of pak choi by regulating its metabolic and transcriptional profiles (Sun et al. 2020; Wang et al. 2025). While transcriptomic and proteomic analyses have revealed carbon and nitrogen metabolism responses in alfalfa (*Medicago sativa L.*) exposed to titanium dioxide nanoparticles (TiO₂ NPs) (Chen et al. 2024). These findings demonstrate the role of multi-omics in dissecting nanomaterial-crop interactions, laying a foundation for subsequent ZnO NMs and soybean quality research.

Although multi-omics technologies have been widely applied in the research on the interaction between nanomaterials and crops, the related studies on ZnO NMs in soybeans mainly focused on physiological and biochemical reactions, stress resistance or toxicology (Jia et al. 2025; Yang et al. 2021). Most existing studies only employed a single application mode and limited concentration gradients, lacking systematic comparison of different material types, application methods and dose effects across the whole soybean growth period. Furthermore, previous multi-omics studies mostly focused on overlapping pathway identification, and few have constructed a complete regulatory cascade from transcript level to protein and metabolite level to systematically explain the quality improvement mechanism of ZnO NMs. Currently, systematic research that clarifies the molecular mechanism of ZnO NMs in regulating soybean quality is highly desired. A systematic comparison of the effects of nanomaterial types, application modes and concentrations, as well as the optimal quality-improving combinations will help the application of ZnO NMs in soybean production (Almendros et al. 2022; Paranimuthu et al. 2026).

To fill these gaps, this study used two ZnO NMs with different size and characteristics, *i.e.* ZnO QDs and ZnO NPs, and employed root/foliar application modes with different concentration gradients to treat soybean, and then focused on their effects on soybean seedling and maturity stages. The optimal treatment groups were selected according to the growth parameters at seedling stage and the yield/quality parameters at mature stage compared with the CK group. Finally, transcriptomics, proteomics and metabolomics were used to comprehensively analyze the soybean grains at mature stage to clarify the quality control mechanism of ZnO NMs. The study tried to associate multi-omics data with phenotypic traits to clarify the regulatory effects and internal mechanisms of ZnO NMs, providing theoretical and technical support for their precise application in high-quality soybean production.

## Materials and methods

### Materials and reagents

Zinc (II) acetate dihydrate (Zn(Ac)_2_·2H_2_O), potassium hydroxide (KOH), ethylene glycol (EG), sodium hypochlorite (NaClO), anthrone, sulfuric acid, and 80% ethanol were purchased from Beijing Sinopharm Chemical Reagent Co., Ltd. (Beijing, China), all of analytical grade. The BCA Protein Content Detection Kit was supplied by Suzhou Comin Biotechnology (Suzhou, China). Soybean seeds (cultivar: Ruixian 181) were obtained from a local agricultural technology extension center (Guiyang, China). The seed germination rate was verified to be ≥ 90% before the experiment to ensure uniformity of experimental materials. Loam soil used for soybean cultivation was collected from farmland in Guiyang City (Guizhou Province, China), with a pH value of approximately 6.6. The concentrations of available nitrogen, phosphorus and potassium in the soil are 73.04 ± 9.65 mg/kg, 24.32 ± 3.46 mg/kg and 98.74 ± 12.41 mg/kg respectively.

### Synthesis and characterization of ZnO NMs

The synthesis of ZnO QDs was according to the previously reported sol method with optimizations (Zhao et al. 2020). Firstly, 16.62 g Zn(Ac)_2_·2H₂O was dissolved in 400 mL absolute ethanol and stirred continuously at 50 °C for 30 min. Separately, 6.18 g KOH was dissolved in 50 mL absolute ethanol, and the solution was stirred ultrasonically at room temperature until complete dissolution. The KOH ethanol solution was then added dropwise to Zn(Ac)₂·2H₂O ethanol solution, with continuous stirring for additional 30 min. During this process, the mixture gradually transitioned from turbid to clear and transparent. The resulting solution was allowed to stand at room temperature for 12 h before being transferred to a rotary evaporator. White precipitates were obtained after evaporation at 50 °C for 6 h. The precipitates were washed several times with absolute ethanol and then centrifuged at 4000 rpm for 15 min. Finally, the precipitate was dried in an oven at 60 °C overnight to yield ZnO QDs.

The synthesis of ZnO NPs was according to the previously reported ethylene glycol method with optimizations (Chieng and Loo 2012). Firstly, 21.95 g Zn(Ac)_2_·2H₂O was dissolved in 100 mL EG and refluxed at 160 °C for 12 h, resulting in the formation of milky-white suspension. The suspension was allowed to cool down to room temperature and then centrifuged at 6000 rpm for 10 min to separate the precipitate from the supernatant. The precipitate was washed several times with absolute ethanol and subsequently centrifuged at 4000 rpm for 15 min. Finally, the obtained precipitate was dried in an oven at 80 °C overnight to obtain ZnO NPs.

The size and morphology of ZnO NMs were recorded by transmission electron microscopy (TEM, JEM-2100, Japan Electronics Co., Ltd.), and the particle size distribution was analyzed by measurement software (Nano measurer 1.2). The fluorescence spectra of ZnO QDs were measured with a fluorescence spectrophotometer (F-7000, Hitachi, Japan). The UV–vis absorption spectra were recorded by using a U-3900 spectrometer (Hitachi, Japan). The X-ray diffraction (XRD, D8, Bruker AXS Germany Ltd.) with a scanning rate of 5°/min in the range of 20–80° was performed to analyze the composition and crystalline structure. The X-ray photoelectron spectroscopy (XPS) spectra were obtained by X-ray photoelectron spectrometer (Thermo Kalpha, USA). The surface functional groups were characterized by Fourier transform infrared spectroscopy (FT-IR, Tensor 27, Bruker Optics, Germany).

### Soybean cultivation and application of ZnO NMs

Soybean seeds were disinfected with 5% NaClO for 5 min, and then thoroughly rinsed with ultrapure water. After soaking seeds for 3 h, sterile seeds were germinated in a dark incubator with an average humidity of 60% at 28 °C for 3 days. After germination, soybean seedlings with uniform growth were selected and transplanted into a gallon pot (4.5 L with 2 kg soil) to grow under natural conditions (temperature ~ 25 °C, humidity ~ 50%), and the soil moisture was maintained at about 75% with a 16 h light/8 h dark photoperiod.

These experiments were conducted to investigate the effects of ZnO QDs and ZnO NPs on the growth and quality of soybean by using two application modes (root application and foliar application) with different application concentrations (0, 5, 10, 20, 50 and 100 mg/kg). One blank control group (CK) and four treatment groups were established: root application of ZnO QDs (R-QD), root application of ZnO NPs (R-NP), foliar application of ZnO QDs (F-QD), and foliar application of ZnO NPs (F-NP). The number suffix of each treatment label indicates the final application concentration of ZnO NMs (mg/kg). All treatments were arranged in a completely randomized design, with at least 10 biological replicates per treatment.

The soil of root application groups (R) was pretreated as follows. Firstly, the stock solutions of two kinds of ZnO NMs were prepared with deionized water, then diluted to the required application concentration of each group (5, 10, 20, 50 and 100 mg/kg), and then mixed with the soil respectively to obtain the soil containing different concentrations of ZnO QDs or ZnO NPs. The soil of the CK and foliar application group (F) were mixed with the same amount of deionized water. In the foliar application groups, ZnO QDs and ZnO NPs were sprayed onto the leaves on the 25th day (V1) and 55th day (V2) after soybean transplanting. Before spraying, ZnO QDs and ZnO NPs suspensions at the required concentration for each group were prepared with deionized water, and sonicated for 20 min to obtain a uniform and stable suspension. Prior to each spraying event, the soil surface of each pot was covered with tinfoil to prevent the ZnO NMs suspension from entering the soil and causing cross-interference with the root application groups. All spraying operations were performed between 8:00 a.m. and 9:00 a.m., when plant stomata are in an open state. Droplets were evenly distributed on the front and back sides of the leaves to avoid dripping. The leaves were kept moist within 12 h after spraying, once a day for 5 consecutive days. The amount of ZnO NMs sprayed twice remained the same, and the total amounts of ZnO NMs in the root application groups and foliar application groups were the same, while the CK group was sprayed with the same volume of deionized water in the same way.

### Determination of growth parameters during soybean seedling stage

Before the second foliar application of ZnO NMs (V2 stage), five soybean plant samples were randomly selected from each treatment group, and the surface of leaves and roots were gently washed with ultrapure water, and the excess water was removed with absorbent paper. The plant height, stem diameter, root parameters, shoot and root biomass (fresh weight and dry weight) of soybean seedlings were measured. The main stem height measured with a tape measure was the plant height of soybean (from the base of the basin soil surface to the growth point of the top of the main stem). The stem diameter of soybean was measured with vernier caliper (10 cm from the base of the basin soil surface). Root parameters (root length, root tip number, root volume, root surface area and root morphology) of soybean plants were analyzed by root scanner (WinRHIZO Pro 2017b, Regent Instruments Inc., Canada). Then the fresh and dry weights of shoot and root parts of soybean were measured by weighing method. The dry weight of the fresh plant sample was quenched at 115 ℃ for 15 min, dried at 70 ℃ for 48 h to constant weight, and then weighed with an electronic balance (Ohaus, Shanghai). The remaining soybean plants grew to maturity according to the original conditions.

### Determination of soybean seed parameters

All soybeans were harvested after growing for about 110 days. Firstly, the soybean yield parameters (the pod number, pod weight, and seed number) of soybean maturity period were determined. Then, the soybean quality parameters (the soluble sugar content and protein content) of soybean seeds were measured separately. Each treatment underwent at least three independent biological replicates.

To determine the protein content of the seeds, approximately 0.1 g fresh soybean grain samples were taken for each treatment, ground into uniform powder in liquid nitrogen, mixed well with 1 mL distilled water, and then homogenized in an ice bath. The samples were then centrifuged at 8000 rpm, 4 °C for 10 min. The supernatant was taken and the protein content was determined using the BCA Protein Content Detection Kit. The experimental steps were carried out in accordance with the instructions of the kit. To determine the content of soluble sugar, fresh soybean seed samples were baked in an oven at 110 °C for 15 min, and then the temperature was adjusted to 70 °C for 48 h until a constant weight was achieved. After the dry sample was ground evenly, it was homogenized with 80% ethanol in a boiling water bath, centrifuged at 10,000 rpm for 10 min, and the supernatant was mixed with anthrone-sulfuric acid solution (2 g anthrone dissolved in 80% H_2_SO_4_ and made up to 1 L) at a ratio of 1:10. After incubation in a boiling water bath for 10 min, the sample was rapidly cooled in an ice bath to terminate the reaction and then allowed to equilibrate to room temperature. The absorbance of the solution was determined at 620 nm using a UV–Vis spectrophotometer (Dong et al. 2023; Sadak et al. 2019).

### Multi-omics analysis of soybean seeds

Through the comprehensive evaluation of the yield and quality parameters of soybean at maturity, the optimal ZnO NMs, application mode, and concentration combination to promote the growth and improve the quality of soybean were determined. Transcriptomics, proteomics and metabolomics analyses were performed on fresh soybean seeds in the optimal group and CK group, with 6 biological replicates in each group.

For transcriptomics analysis, total RNA was extracted from soybean seed samples. After quality validation, cDNA libraries were constructed and sequenced on the Illumina NovaSeq 6000 platform. Raw reads were quality-controlled by Fastp (v0.23.2) to remove adapters, undetermined bases and low-quality reads (Phred score < 20). Clean reads were aligned to the *Glycine max* reference genome (v2.1) via HISAT2 (v2.2.1), and gene expression was quantified as FPKM using StringTie (v2.0.0). Differential expression analysis was performed with DESeq2 (v1.20.0), with |log_2_FC|> 1 and the false discovery rate (FDR) < 0.05 as screening thresholds for differentially expressed genes (DEGs). Gene Ontology (GO) and Kyoto Encyclopedia of Genes and Genomes (KEGG) pathway enrichment analyses were conducted by clusterProfiler (v4.0.2) with Benjamini-Hochberg (BH) correction.

For proteomics analysis, total protein was extracted from seed samples, digested with trypsin, and analyzed via data-independent acquisition (DIA) on a UHPLC coupled Thermo Orbitrap mass spectrometer. Raw data were processed by Spectronaut (v15.0) for protein identification and label-free quantification. Differentially expressed proteins (DEPs) were screened with thresholds of |log_2_FC|> 1.5 and FDR < 0.05. GO and KEGG functional enrichment analyses were performed using clusterProfiler (v4.0.2).

For metabolomics analysis, metabolites were extracted from seed samples with methanol. Non-targeted metabolomic profiling was conducted on a UHPLC-Orbitrap MS system in both positive and negative electrospray ionization modes. Raw data were processed by the R package XCMS (v3.18.0) for peak alignment, retention time correction and peak area quantification. Metabolites were annotated by matching accurate mass and MS/MS spectra against HMDB and KEGG databases. Partial least squares discriminant analysis (PLS-DA) was performed for group discrimination. Differentially abundant metabolites (DAMs) were screened with variable importance in projection (VIP) > 1 and *P* < 0.05 (t-test). KEGG pathway enrichment analysis was conducted via MetaboAnalyst (v5.0).

The detailed descriptions of the procedures for transcriptomics, proteomics and metabolomics analyses were included in the Supplementary Information.

### Validation of key genes via qRT-PCR analysis

Total RNA was extracted from fresh soybean seeds of CK and R‑QD‑50 groups using Trizol reagent (BOLAZ, China), and its concentration and purity were determined with a microspectrophotometer (GE, USA). First‑strand cDNA was synthesized from 1 μg of total RNA using All‑in‑one RT SuperMix for qPCR (BOLAZ, China) in a 20 μL reaction system, with incubation at 50 ℃ for 10 min followed by 85 ℃ for 5 s. qRT‑PCR was performed on an FQD‑96A fluorescent quantitative PCR system (Bori Technology, China) using 2 × Universal SYBR Green qPCR Master Mix (BOLAZ, China) in a 20 μL reaction volume. Specific primers for two target genes (*PAL* and *C4H*) and the reference gene *TUBB3* were synthesized by Jiangsu Bioserve Biotechnology Co., Ltd., with primer sequences listed in Table S1. The amplification procedure consisted of pre‑denaturation at 95 ℃ for 2 min, 35 cycles of 95 ℃ for 15 s, 60 ℃ for 30 s and 72 ℃ for 30 s, and the subsequent melting curve analysis from 55 ℃ to 90 ℃ to confirm amplification specificity. Each sample was analyzed in three technical replicates, and the relative expression levels of target genes were calculated using the 2^−ΔΔCt^ method.

### Multi-omics integration and network analysis

Matched transcriptomic, proteomic and metabolomic profiles from 12 soybean seed samples (6 biological replicates each for the CK and R-QD-50 groups) were used to construct the cross-omics molecular association network. Candidate features included all differentially expressed transcripts, proteins and metabolites, as well as features annotated to phenylpropanoid biosynthesis, flavonoid/isoflavonoid biosynthesis, butanoate metabolism, and taurine and hypotaurine metabolism.

Following missing value imputation, log_2_(x + 1) transformation and low-variance feature filtering, Spearman rank correlation coefficients with two-sided *P* values were calculated exclusively for inter-omics feature pairs (transcript-protein, transcript-metabolite, protein-metabolite). A hierarchical threshold screening strategy was applied from strict to relaxed criteria, and the strictest threshold set (|rho|≥ 0.90, *P* ≤ 0.002) was adopted in this study. Qualified edges were ranked by correlation strength and significance, and the top 900 cross-omics associations were retained to ensure network interpretability. For intuitive and readable visualization, a representative sub-network centered on phenylpropanoid/flavonoid metabolism with the strongest correlations was displayed in the main text.

In the network, nodes represent individual transcripts, proteins and metabolites, while edges represent sample-level statistical associations classified as positive or negative correlations. Core pathway-related features were defined as focus nodes for visualization of the representative subnetwork. All network analyses were implemented in Python (v3.10) using pandas, NumPy, SciPy and NetworkX packages. Detailed analytical protocols, full parameter settings and complete network statistics are provided in the Supplementary Information. It should be noted that the identified associations reflect statistical correlations rather than direct physical interactions or causal regulatory relationships.

### Statistical analysis

The statistical analysis of soybean seedling growth parameters and mature grain parameters in this study was conducted using IBM SPSS Statistics 26.0 software (IBM Corp., Armonk, NY, USA), with a significance level set at *P* < 0.05. Graphs were plotted using Origin 2023. All data were presented as the mean ± standard error (SE).

The replication strategies for different experiments were specified as follows. For phenotypic experiments, at least 10 biological replicates (individual plants) were included for each treatment, and 3 technical replicates were performed for each sample measurement. For multi-omics assays (transcriptomics, proteomics and metabolomics), 6 independent biological replicates were uniformly set for each group to ensure the consistency and comparability of cross-omics integration analysis. Pooled quality control (QC) samples were inserted into the metabolomics detection sequence to monitor instrumental stability and correct for batch effects. For qRT-PCR validation, 3 biological replicates and 3 technical replicates were included for each target gene.

Before formal statistical testing, assumption diagnostics were performed to reduce analytical bias. The Shapiro–Wilk test (α = 0.05) was used to assess normality; non-normal datasets were log-transformed via ln(x + 1), and normality was reconfirmed after transformation (Shapiro and Wilk 1965). Levene’s test (α = 0.05) was used to evaluate variance homogeneity (Brown and Forsythe 1974). For data satisfying normality and homogeneous variance, one-way ANOVA coupled with Tukey HSD pairwise comparisons was adopted, which strictly controls FWER at α = 0.05 for balanced designs. Welch’s ANOVA with Games–Howell post-hoc tests was applied when heteroscedasticity existed. Partial η_p_^2^, 95% CIs and Cohen’s d were calculated to quantify effect magnitudes, where d > 0.8 = large effect, 0.5 ≤ d ≤ 0.8 = moderate effect, d < 0.5 = small effect.

Three-way ANOVA was implemented to evaluate main and interactive effects of nanomaterial type, application mode and concentration, with full factorial models retaining all two-way and three-way interaction terms. Partial η_p_^2^ was calculated to estimate factor effect strength.

Pearson correlation analysis was performed to explore linear associations between seedling and mature-stage traits. The BH procedure was adopted to control FDR at *q* < 0.05 and mitigate false positives from multiple correlation tests.

Principal component analysis (PCA) was carried out based on seven core indicators: seedling traits (plant height, stem diameter, root dry weight), yield traits (pod weight per plant, seed number per plant), grain quality traits (soluble protein, soluble sugar). Raw data were Z-score standardized before analysis. Dataset suitability was verified via Kaiser–Meyer–Olkin (KMO) test (threshold > 0.6) and Bartlett’s sphericity test (*P* < 0.05). Components with cumulative explained variance ≥ 85% were retained. Comprehensive treatment scores were calculated via weighted summation, with each component’s explained variance as weighting coefficients.

## Results and discussion

### Preparation and characterization of ZnO NMs

In this study, two kinds of ZnO NMs, *i.e.* ZnO QDs and ZnO NPs were prepared. These ZnO NMs were then characterized by different modes. Detailed characterization diagrams are shown in the supplementary materials (Fig. S1, S2).

TEM results show that ZnO QDs are nearly spherical in shape and uniform in morphology (Fig. S1a). The average particle size of ZnO QDs is about 5.56 nm (Fig. S1b). The shape of ZnO NPs is also uniform and close to spherical (Fig. S2a). The average particle size of ZnO NPs is about 29.68 nm, much larger than that of ZnO QDs (Fig. S2b). The fluorescence spectrum shows that ZnO QDs exhibit the maximum excitation wavelength of 370 nm, and the emission peak is at about 575 nm (Fig. S1c). The inset image in Fig. S1c illustrates that ZnO QDs aqueous solution is clear and transparent under sunlight (left), while it shows bright yellow fluorescence under ultraviolet light (right), which is consistent with the reported characteristics of ZnO QDs (Chen et al. 2021). UV–vis absorption spectrum shows that ZnO QDs exhibit a broad UV absorption band with no distinct sharp peak, a typical signature of quantum confinement effects in ZnO QDs (Fig. S1c) (Wong et al. 2016). ZnO NPs show a strong near‑band‑edge absorption peak at 357 nm with a broad absorption range of 360–380 nm (Fig. S2c).

Then, XRD spectra were employed to validate the structure and purity of ZnO QDs and ZnO NPs (Fig. S1d, S2d). Compared with the reference spectra for standard ZnO (no. 36–1451) reported by the Joint Committee on Powder Diffraction Standards (JCPDS), the recorded spectra are extremely consistent with the standard JCPDS data card (Fig. S1d, S2d). The main diffraction peaks of ZnO QDs and ZnO NPs correspond to the (100) (002) (101) crystal plane, and the characteristic peaks are located at 31.85, 34.75, 36.28, 47.67, 56.72, 62.97, 68.25° (Fig. S1d, S2d). The structures of the two kinds of ZnO NMs remain intact, forming a typical hexagonal wurtzite structure. Due to the quantum dot size effect, the diffraction peaks of ZnO QDs become wider and the intensity decreases, resulting in a slight peak shift (Fig. S1d), while the diffraction peaks of ZnO NPs become clearer with the increase of intensity (Fig. S2d). Sharp diffraction peaks indicate that ZnO NPs have good crystallization quality, while the absence of additional redundant peaks clearly shows that the synthesized sample is pure and free of impurities (Fig. S2d). XPS spectra show that the two kinds of ZnO NMs have characteristic peaks in Zn 2p and O 1 s, which confirm that the samples are pure ZnO without other impurities (Fig. S1e, 2e). The FTIR spectra show that both ZnO NMs contain Zn–O lattice vibration peaks (400–600 cm^−1^) and surface hydroxyl/adsorbed water peaks (3200–3600 cm^−1^, 1620–1640 cm^−1^) (Fig. S1f, S2f). However, the broadening of Zn–O peak of ZnO QDs is weakened, and the hydroxyl/defect peak is stronger (Fig. S1f). Zn–O peak of ZnO NPs is sharper, and the surface correlation peak is weaker (Fig. S2f). The results of the above characterizations finally verify the successful synthesis of ZnO QDs and ZnO NPs.

### Effect of ZnO NMs application on the growth of soybean seedlings

**Fig. 1:**
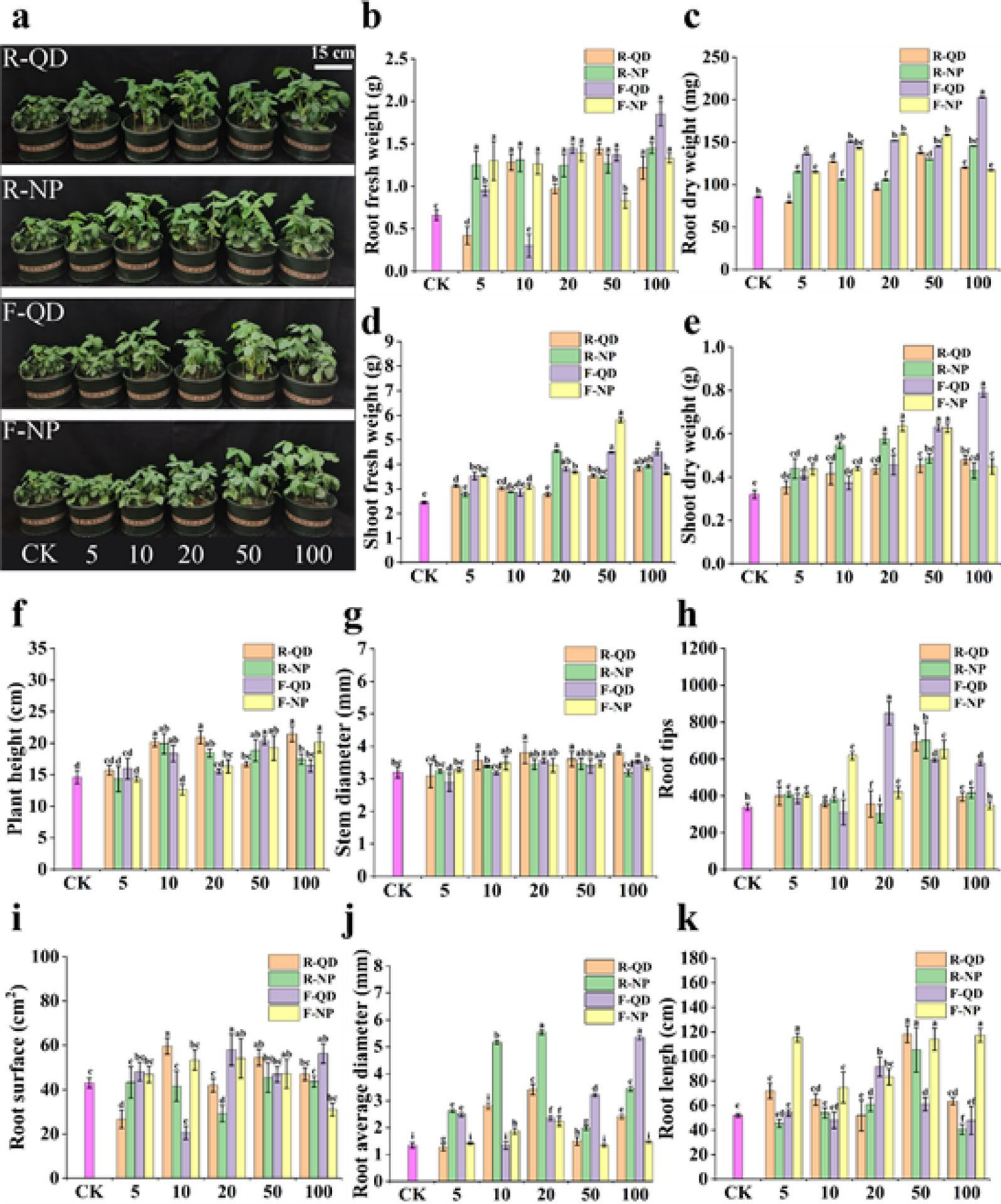
Effect of ZnO NMs application on the growth of soybean seedlings. **a** Growth status of soybean seedling stage. **b** The root fresh weight. **c** The root dry weight. **d** The shoot fresh weight. **e** The shoot dry weight. **f** The plant height. **g** The stem diameter. **h** The root tips. **i** The root surface. **j** The root average diameter. **k** The root length. Data are presented as mean ± SE of 3 biological replicates. Different lowercase letters indicate significant differences among treatments (one-way ANOVA, Tukey HSD test, *P* < 0.05)

Phenotypic display of soybean seedling stage reveals ZnO NMs significantly promoted soybean seedling growth, with effects dependent on nanomaterial type, application mode, and concentration (Fig. 1a). Root application of 100 mg/kg ZnO QDs (R-QD-100) and root application of 50 mg/kg ZnO NPs (R-NP-50) induced vigorous seedling growth. Foliar application of ZnO QDs (F-QD) exhibited a stronger growth-promoting effect at high concentrations (50 mg/kg, 100 mg/kg), while the growth-promoting effect of foliar application of ZnO NPs (F-NP) increased with increasing concentration (Fig. 1a). Similar concentration-dependent promotion effects of ZnO NMs on crop growth have been reported (Pan et al. 2026).

Biomass accumulation was significantly affected by nanomaterial type, application mode, and concentration (*P* < 0.05) (Fig. 1b–e). For root biomass, F-QD-100 treatment showed the highest values (fresh weight: 1.85 g, 2.14 × that of CK; dry weight: 202.87 mg, 136.7% higher than CK, *P* < 0.05) (Fig. 1b, c). In contrast, R-QD-5 treatment inhibited root biomass accumulation (fresh weight: 63.6% of CK; dry weight: 92.4% of CK) (Fig. 1b, c). For shoot fresh weight, F-NP-50 treatment showed the highest value (5.81 g), which was 138.1% higher than that of CK (*P* < 0.05), followed by R-NP-20 (4.54 g, 86.1% higher than CK, *P* < 0.05) (Fig. 1d). For shoot dry weight, F-QD-100 treatment exhibited the highest value (0.79 g, 146.9% higher than CK, *P* < 0.05), followed by F-NP-20 (0.64 g, twice that of CK, *P* < 0.05) (Fig. 1e).

Plant height and stem diameter are key indicators reflecting the regulatory effects of ZnO NMs on soybean seedling growth (Fig. 1f, g). R-QD-100 (21.47 cm, 47.1% higher than CK, *P* < 0.05) and F-NP-100 (20.13 cm, 37.9% higher than CK, *P* < 0.05) treatments showed the highest plant height (Fig. 1f). For stem diameter, R-QD (except R-QD-5), F-QD-20, and F-QD-100 treatments were optimal (3.54–3.81 mm, *P* < 0.05), while F-QD-5 treatment was the lowest (2.89 mm, 10.72% lower than CK, *P* < 0.05) (Fig. 1g).

Root parameters are closely correlated with biomass accumulation (Fig. 1h–k). For root tip number, F-QD-20 treatment showed the highest value (847, 149.1% higher than CK, *P* < 0.05), followed by R-NP-50 (701, 106.2% higher than CK, *P* < 0.05) (Fig. 1h). For root surface area, R-QD-10 treatment exhibited the highest value (59.57 cm^2^, 38.3% higher than CK, *P* < 0.05), while F-QD-10 treatment showed the lowest (20.46 cm^2^, 47.6% of CK, *P* < 0.05) (Fig. 1i). For average root diameter, R-NP-20 (5.54 mm, 4.13 × that of CK, *P* < 0.05) and F-QD-100 (5.35 mm, 3.99 × that of CK, *P* < 0.05) treatments were the optimal (Fig. 1j). For root length, R-QD-50 treatment was the longest (118.17 cm, 127.8% higher than CK, *P* < 0.05) (Fig. 1k).

Collectively, ZnO NMs promoted soybean seedling growth by improving root morphology and biomass accumulation. ZnO QDs exhibited greater root growth-promoting advantage than ZnO NPs. Root application favored root development, while foliar application enhanced the aboveground biomass accumulation. Moderate to high concentration of ZnO NMs was the optimal condition for seedling growth, laying a solid foundation for improved mature yield and grain quality.

### Effect of ZnO NMs application on soybean mature grains

**Fig. 2:**
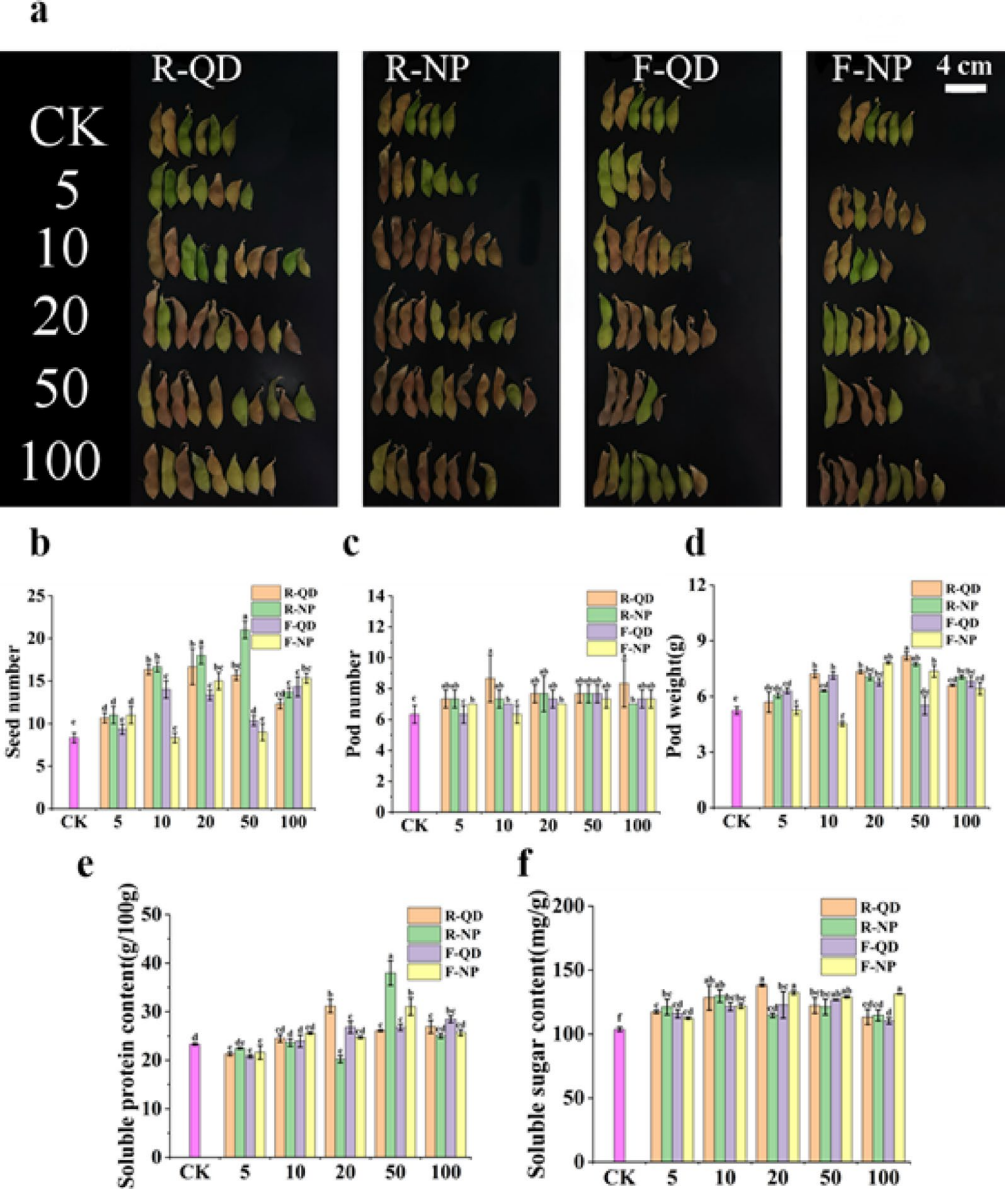
Effect of ZnO NMs application on soybean mature grains. **a** Harvesting situation of mature soybean. **b** The seed number. **c** The pod number. **d** The pod weight. **e** The soluble protein content of soybean seeds. **f** The soluble sugar content of soybean seeds. Data are presented as mean ± SE of 3 biological replicates. Different lowercase letters indicate significant differences among treatments (one-way ANOVA, Tukey HSD test, *P* < 0.05)

ZnO NMs effectively improved soybean yield and grain quality at maturity, with treated pods outperforming CK (Fig. 2a). For R-QD and R-NP treatments, pod number and plumpness increased with the treatment concentration (a slight decrease at 100 mg/kg), while F-QD and F-NP treatments had weaker effects on pod development (Fig. 2a).

Yield parameters were significantly affected by ZnO NM treatments (*P* < 0.05) (Fig. 2b–d). R-NP-50 treatment showed the highest seed number per plant (Fig. 2b), while R-QD-10 treatment had the highest pod number per plant (Fig. 2c). For pod weight per plant, R-QD-50 treatment was optimal (8.18 g, 56.1% higher than CK, *P* < 0.05), and F-NP-10 treatment was the lowest (4.53 g, 13.5% lower than CK, *P* < 0.05) (Fig. 2d).

ZnO NMs treatments improved soybean grain quality (Fig. 2e–f). R-NP-50 treatment showed the highest protein content (37.96 g/100 g, 63.0% higher than CK, *P* < 0.05) (Fig. 2e). R-QD-20 treatment exhibited the highest soluble sugar content (138.25 mg/g, 33.3% higher than CK, *P* < 0.05) (Fig. 2f).

Root application outperformed foliar application, likely due to the longer nutrient supply duration. ZnO QDs favored pod traits, while ZnO NPs promoted seed yield and protein accumulation, indicating specific regulatory effects of different ZnO NMs types. Moderate to high concentration of ZnO NMs (20 mg/kg, 50 mg/kg) were the optimal condition, with 100 mg/kg slightly reducing root application performance. The slightly decreased performance at 100 mg/kg is presumably related to phytotoxicity induced by excessive Zn^2+^ released from ZnO NMs (Sheteiwy et al. 2021). Firstly, the root morphology at seedling stage showed typical zinc toxicity phenotypes: the number of root tips decreased significantly, and root elongation was inhibited. Secondly, all growth, yield and quality indicators showed a typical “low-dose promotion, high-dose inhibition” hormesis effect, which is consistent with the dose–response characteristics of heavy metal phytotoxicity. However, this study did not directly determine physiological and biochemical indicators such as tissue zinc content and reactive oxygen species levels, so the specific toxicity mechanism still needs further verification.

### Statistical analysis and optimal regime determination

Prior to one-way ANOVA, Shapiro–Wilk normality tests returned* P* > 0.05 for all groups, meaning the null hypothesis of normal distribution could not be rejected. Levene’s test also yielded *P* > 0.05 across all indicators, showing no significant heteroscedasticity among treatments.

Parametric assumptions were fully satisfied, so standard one-way ANOVA followed by Tukey HSD pairwise comparisons was conducted across five treatments (CK, R-QD, R-NP, F-QD, F-NP). Standardized effect sizes and 95% confidence intervals were calculated to quantify treatment efficacy. Partial η_P_^2^ values from one-way ANOVA ranged from 0.62 to 0.85, indicating extremely strong treatment effects. Cohen’s d values for the core R-QD-50 versus CK comparison were 0.965 (root length), 2.114 (pod weight per plant), and 2.090 (soluble sugar content), all corresponding to large beneficial effects. The 95% CIs for raw mean differences between R-QD-50 and CK were [60.453, 87.701] cm (root length), [6.510, 7.495] g (pod weight per plant), and [118.223, 129.650] mg/g (soluble sugar content).

Three-way ANOVA further quantified factor contributions. Application mode exerted the strongest main effect (η_P_^2^ = 0.76), followed by nanomaterial type (η_P_^2^ = 0.68) and concentration (η_P_^2^ = 0.59). All two-way and three-way interaction terms showed no statistical significance (*P* > 0.05), and thus only main effects were interpreted without further stratification analysis. Root application significantly outperformed foliar spraying (*P* < 0.05). ZnO QDs mainly promoted pod and root growth, while ZnO NPs facilitated seed and protein accumulation; moderate-to-high concentrations generated optimal agronomic performance (*P* < 0.05).

Pre-PCA suitability tests showed a KMO value of 0.6914, exceeding the 0.6 adequacy threshold. Bartlett’s sphericity test was significant (χ^2^ = 26.9082, df = 6, *P* = 0.00015066), confirming strong inter-variable correlations. Two principal components were extracted, accounting for over 85% cumulative explained variance and capturing most phenotypic variation. Weighted comprehensive scores produced the treatment efficacy ranking: R-QD > R-NP > F-QD > F-NP > CK, providing statistical evidence for preliminary optimal treatment screening.

To further validate the rationality of the optimal regime, dose–response validation verified the performance of the top-ranked group. All core indicators continuously improved from 5 mg/kg to 50 mg/kg, consistent with the low-dose stimulatory property of nanomaterials. Promotional effects weakened markedly at 100 mg/kg, reflecting high-dose inhibition. The 20 mg/kg treatment only improved soluble sugar without obvious yield promotion, whereas 50 mg/kg achieved the highest comprehensive benefit per unit input, avoiding material waste and environmental risks from high concentrations.

Collectively, ZnO NM regulatory effects on soybean growth, yield and quality were primarily governed by application mode, followed by nanomaterial type and concentration. Combined with PCA ranking and dose-gradient verification, R-QD-50 was identified as the optimal regime and selected for subsequent multi-omics analysis to resolve the molecular mechanisms of ZnO NM-mediated soybean quality improvement. This optimized strategy also provides theoretical references for ZnO NM application in other crop species.

### Transcriptome sequencing and analysis of soybean seeds

**Fig. 3:**
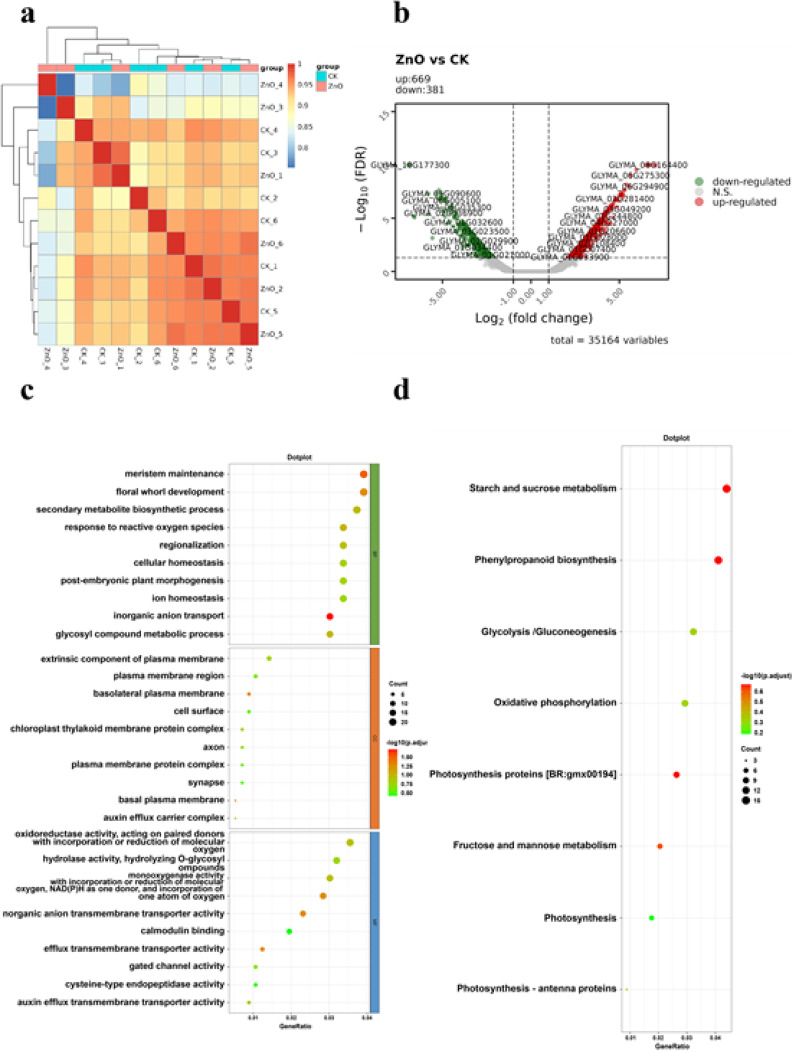
Transcriptomic analysis of soybean seeds from R-QD-50 and CK groups. **a** Pearson correlation heatmap (values in grids: the pearson correlation coefficient ranging from 0 to 1). **b** Volcano plot of DEGs (red dots: significantly upregulated genes, green dots: significantly downregulated genes, gray dots: non-significantly changed genes). **c** GO enrichment bubble plot of DEGs. Three panels from top to bottom correspond to GO Biological Process (BP), Cellular Component (CC) and Molecular Function (MF) respectively; X-axis indicates GeneRatio (higher value = higher enrichment degree); bubble size represents the number of enriched DEGs (Count); bubble color shows enrichment significance (redder color = higher -log_10_(*P*. adjust) = higher statistical significance). **d** KEGG enrichment bubble plot of DEGs. X-axis indicates GeneRatio (higher value = higher enrichment degree); bubble size represents the number of enriched DEGs (Count); bubble color shows enrichment significance (redder color = higher -log_10_(*P*. adjust) = higher statistical significance). DEGs were screened with |log_2_FC|> 1 and *P* < 0.05

To explore the transcriptional regulatory mechanisms underlying ZnO NMs-mediated soybean quality improvement, transcriptomic analysis was performed on fresh soybean seeds in the R-QD-50 and CK groups. After quality control, clean data exhibited Q20 values of 98.19%-98.71%, Q30 values of 94.52%-96.10%, and uniquely mapped reads of 91.93%-96.96%. Intra-group Pearson correlation coefficients exceeded 0.95 with clear clustering (Fig. 3a), confirming data reliability and repeatability. A total of 44,266 mRNAs were detected, and 1050 DEGs were identified (|log_2_FC|> 1, *P* < 0.05), including 669 upregulated and 381 downregulated genes, indicating R-QD-50 induced extensive transcriptional activation in soybean seeds (Fig. 3b).

GO enrichment analysis annotated DEG functions into three categories: biological process (BP), cellular component (CC), and molecular function (MF) (Fig. 3c) (Haidar et al. 2024). BP was the primary focus, while CC and MF provided supporting mechanistic information. For BP, meristem maintenance and floral whorl development showed both the highest statistical significance and GeneRatio; inorganic anion transport exhibited the highest significance among transport-related terms. CC terms were mainly distributed in plasma membrane-related components (*e.g.*, extrinsic component of plasma membrane and plasma membrane region) and chloroplast thylakoid membrane protein complex. MF terms included oxidoreductase, hydrolase, and ion transporter activities, corresponding to catalytic reactions and ion transport processes underlying BP functions.

KEGG enrichment analysis mapped DEGs to biological pathways, identifying eight significantly enriched pathways (*P*. adjust < 0.05, Fig. 3d). Among them, Starch and sucrose metabolism and Phenylpropanoid biosynthesis showed both the highest statistical significance (red color) and GeneRatio (rightmost position), representing the core transcriptional response pathways directly linked to soybean seed quality formation. Additionally, Photosynthesis proteins exhibited high significance, while energy-related (Glycolysis/Gluconeogenesis, Oxidative phosphorylation), carbohydrate-related, and other photosynthesis-related pathways were also enriched, forming a coordinated regulatory network. The upregulation of Starch and sucrose metabolism directly explained the increased soluble sugar, while activated Phenylpropanoid biosynthesis, the core upstream pathway of flavonoid and isoflavone biosynthesis in plants, contributed to the accumulation of quality-related secondary metabolites (e.g., isoflavones, lignin) (Paranimuthu et al. 2026; Quan et al. 2026).

These findings indicate that ZnO NMs extensively regulate gene expression related to development, metabolism, and stress responses in soybean seeds. The activated transcriptional program provides a fundamental basis for improved seed quality through coordinated metabolic and physiological changes.

### DIA-based proteomics analysis of soybean seeds

**Fig. 4:**
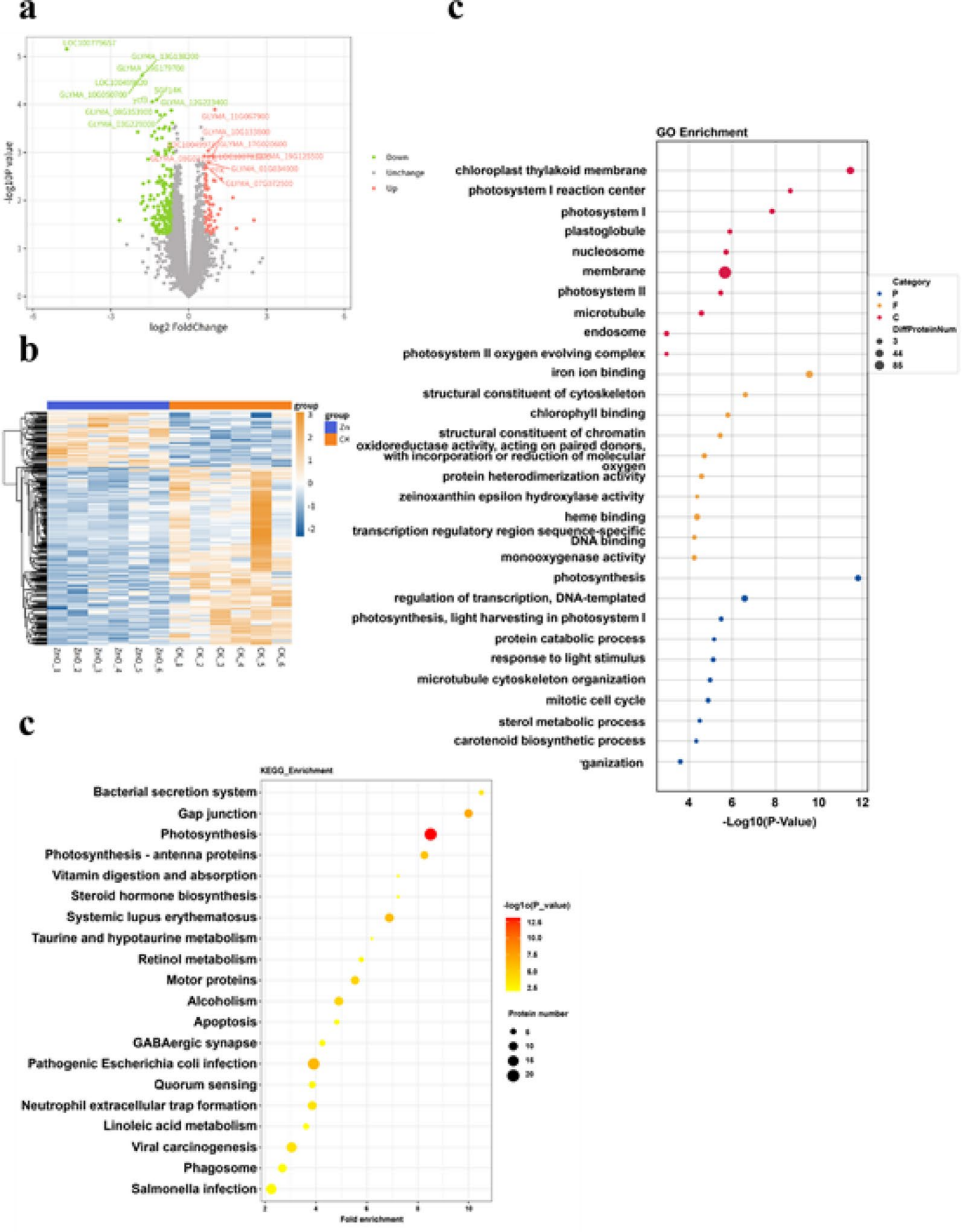
Proteomic analysis of soybean seeds from R-QD-50 and CK groups. **a** Volcano plot of DEPs (red dots: significantly upregulated proteins, blue dots: significantly downregulated proteins, gray dots: no significant expression change). **b** Hierarchical clustering heatmap. The top color bar indicates sample grouping where the color gradient represents relative protein expression (orange: high expression, blue: low expression). **c** GO enrichment bubble plot of DEPs (Blue dots: Biological Process (BP); orange dots: Molecular Function (MF); red dots: Cellular Component (CC); X-axis indicates -log_10_(*P*-value): higher value = higher enrichment significance). **d** KEGG enrichment bubble plot of DEPs. X-axis indicates Fold enrichment (higher value = higher enrichment degree); bubble size represents the number of enriched DEPs; bubble color shows enrichment significance (redder color = higher -log_10_(*P*-value) = higher statistical significance). DEPs were screened with |log_2_FC|> 1.5 and *P* < 0.05

To further explore the post-transcriptional regulatory responses to ZnO NMs treatment, DIA-based proteomic analysis was performed on fresh soybean seeds in the R-QD-50 and CK groups. Using thresholds of *P* < 0.05 and |log_2_FC|> 1.5, 435 DEPs were identified, including 102 upregulated and 333 downregulated proteins (Fig. 4a). Hierarchical clustering showed clear separation between ZnO NMs and CK groups with high intra-group similarity, indicating a distinct proteomic response (Fig. 4b).

GO enrichment showed DEPs were highly enriched in chloroplast thylakoid membrane, photosystem I/II (CC), chlorophyll/iron ion binding and hydrolase activity (MF), as well as photosynthesis and microtubule cytoskeleton organization (BP) (Fig. 4c). KEGG enrichment analysis confirmed that the Photosynthesis pathway was the most significantly enriched, consistent with GO results (Fig. 4d). The Photosynthesis pathway was dominated by downregulated DEPs, whereas amino acid biosynthesis and sterol metabolism pathways were enriched with upregulated DEPs.

Proteomic data reveal that the downregulated proteins were mainly enriched in light-harvesting antenna systems and photosystem reaction centers, whereas those related to carbon fixation and amino acid biosynthesis were maintained or even upregulated. Collectively, this moderate downregulation of photosynthetic proteins in soybean seeds is not an overall metabolic inhibition caused by toxicity, but rather a typical adaptive response to ZnO NMs exposure. As reproductive organs with inherently weak photosynthetic capacity, soybean seeds undergo adaptive proteomic remodeling. They reduce energy input for light harvesting and reallocate intracellular resources toward the anabolic metabolism of quality-related substances (e.g., storage proteins, isoflavones, and soluble sugars). Meanwhile, ZnO NMs significantly promote root development and nutrient absorption, enhancing source-sink transport efficiency from vegetative organs to grains. This allows seeds to obtain sufficient carbon and nitrogen from the maternal plant, further supporting cellular homeostasis and sustained anabolic growth (Ruotolo et al. 2018). Ultimately, this resource reallocation strategy directly promotes the accumulation of nutritional and functional components, which is highly consistent with the significant increases in seed protein, soluble sugar, and secondary metabolite contents observed in our phenotypic analysis.

### UHPLC-MS/MS metabolomics analysis of soybean seeds

**Fig. 5:**
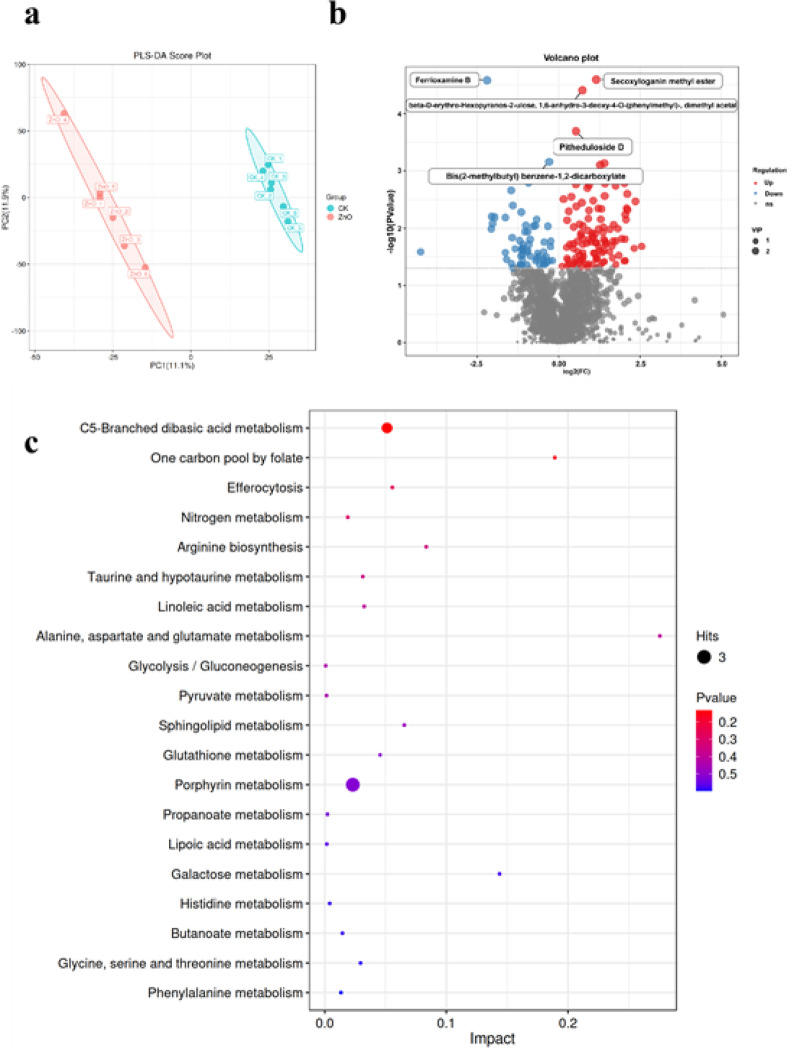
Metabolic changes in soybean seeds from R-QD-50 and CK groups. **a** Score plot of partial least squares discriminant analysis (PLS-DA). **b** Volcano plot of DAMs (red dots: significantly upregulated metabolites, blue dots: significantly downregulated metabolites, gray dots: metabolites with no significant abundance change). **c** KEGG enrichment bubble plot of DAMs. X-axis indicates pathway impact value from topological analysis; bubble size represents the number of enriched DAMs (Hits); bubble color shows enrichment significance (red = lower *P* value, higher significance; blue = higher *P* value, lower significance). DAMs were screened with VIP > 1 and *P* < 0.05

To explore the metabolic reprogramming triggered by ZnO NMs treatment, UHPLC-MS/MS-based non-targeted metabolomic analysis was performed on fresh soybean seeds in the R-QD-50 and CK groups. PLS-DA showed clear separation between ZnO NMs-treated and CK groups with tight intra-group clustering, indicating distinct metabolic profiles (Fig. 5a). A total of 2567 metabolites were identified. Based on *P* < 0.05 and VIP > 1, 170 DAMs were obtained, including 108 upregulated and 62 downregulated metabolites, demonstrating a dominant promotion effect of ZnO treatment on soybean seeds metabolism (Fig. 5b).

KEGG enrichment analysis showed DAMs were mainly enriched in amino acid metabolism and energy metabolism, with C5-branched dibasic acid metabolism, nitrogen metabolism, and arginine biosynthesis as core pathways (Fig. 5c). Nitrogen metabolism facilitated ammonia assimilation and amino acid precursor synthesis (Fig. S3). Arginine biosynthesis was activated, with glutamate as a key precursor for L-arginine production (Fig. S4).

The widespread reprogramming of amino acid and nitrogen metabolism reflected improved nutrient uptake and utilization efficiency under ZnO NMs treatment, providing sufficient precursors for protein and secondary metabolite synthesis.

### Integrated analysis of transcriptomics and proteomics

**Fig. 6:**
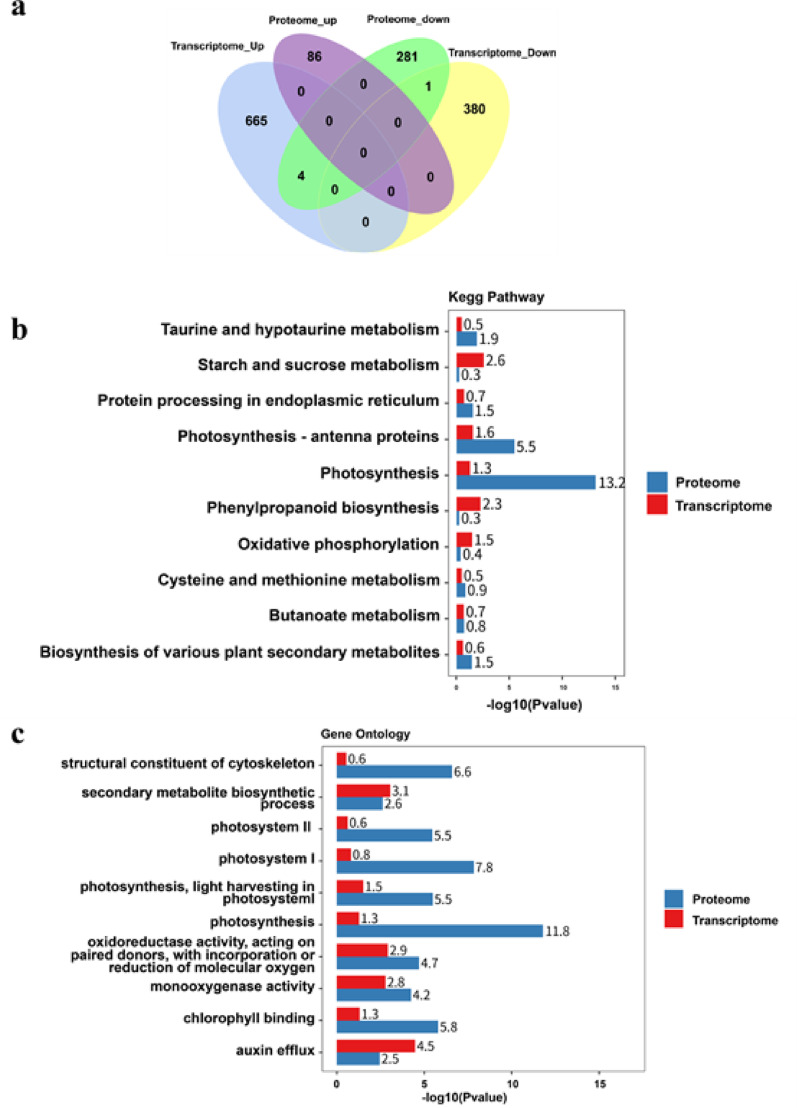
Integrated analysis of transcriptomics and proteomics on soybean seeds from R-QD-50 and CK groups. **a** Venn diagram showing the overlap between DEGs and DEPs. **b** KEGG pathway enrichment analysis of DEGs and DEPs. **c** GO enrichment analysis of DEGs and DEPs. In the enrichment plots, bar length represents enrichment significance as -log_10_(*P*), blue bars: transcriptomic DEGs, red bars: proteomic DEPs

Differential expression analysis revealed distinct transcriptional-translational regulation patterns (Fig. 6a). The transcriptome featured 665 uniquely upregulated and 380 uniquely downregulated genes. The proteome exhibited 86 uniquely upregulated and 281 uniquely downregulated proteins. Only 4 molecules showed synchronous upregulation; no shared downregulation was detected. This substantial divergence reflects widespread transcriptional-translational decoupling, a common regulatory phenomenon in plant responses to environmental stimuli, indicating that post-transcriptional and post-translational modifications (*e.g.*, protein stability and translational efficiency) strongly shape the final proteomic landscape (Kumar et al. 2025; Ruotolo et al. 2018).

KEGG pathway analysis further confirmed functional divergence (Fig. 6b). The proteome was dominated by photosynthetic pathways, with Photosynthesis (-log_10_(*P*) = 13.2) and Photosynthesis-antenna proteins (-log_10_(*P*) = 5.5) as the most significant. The transcriptome was mainly enriched in starch and energy-related metabolic pathways (Fig. 6b).

GO enrichment analysis demonstrated divergent profiles between omics layers (Fig. 6c). The proteome was significantly enriched in photosynthesis-related processes (-log_10_(*P*) = 11.8), photosystem I/II assembly (-log₁₀(*P*) ≥ 5.5), and structural constituent of cytoskeleton (-log_10_(*P*) = 6.6). The transcriptome was most prominently enriched in auxin efflux (-log_10_(*P*) = 4.5). Moderate shared enrichment was observed for secondary metabolite biosynthesis (Fig. 6c).

The obvious transcriptional-translational decoupling revealed a layered regulatory strategy. The proteome maintained photosynthetic function, while the transcriptome dominated signal transduction and metabolic regulation.

### Integrated three‑omics analysis of shared pathways and cross‑omics regulatory networks

Based on transcriptomic and proteomic integration, 10 core regulatory pathways were identified. Combined with 29 main metabolic pathways from metabolomics, three shared pathways across three omics were screened: taurine and hypotaurine metabolism, butanoate metabolism, and phenylpropanoid biosynthesis (Table S2). Consistent with previous multi-omics studies, the pathway shared by transcriptome, proteome and metabolome was identified as the core regulatory chain of ZnO NMs-mediated soybean quality regulation (Wang et al. 2024). Only slight changes were observed in butanoate metabolism at the metabolome level, with no effective linkage to transcriptome and proteome. In taurine and hypotaurine metabolism, taurine content increased by 40% (*P* < 0.05), and the CSAD (Cysteine Sulfinic Acid Decarboxylase) gene showed an upregulation trend (|log_2_FC|= 0.87, *P* = 0.07).

**Fig. 7:**
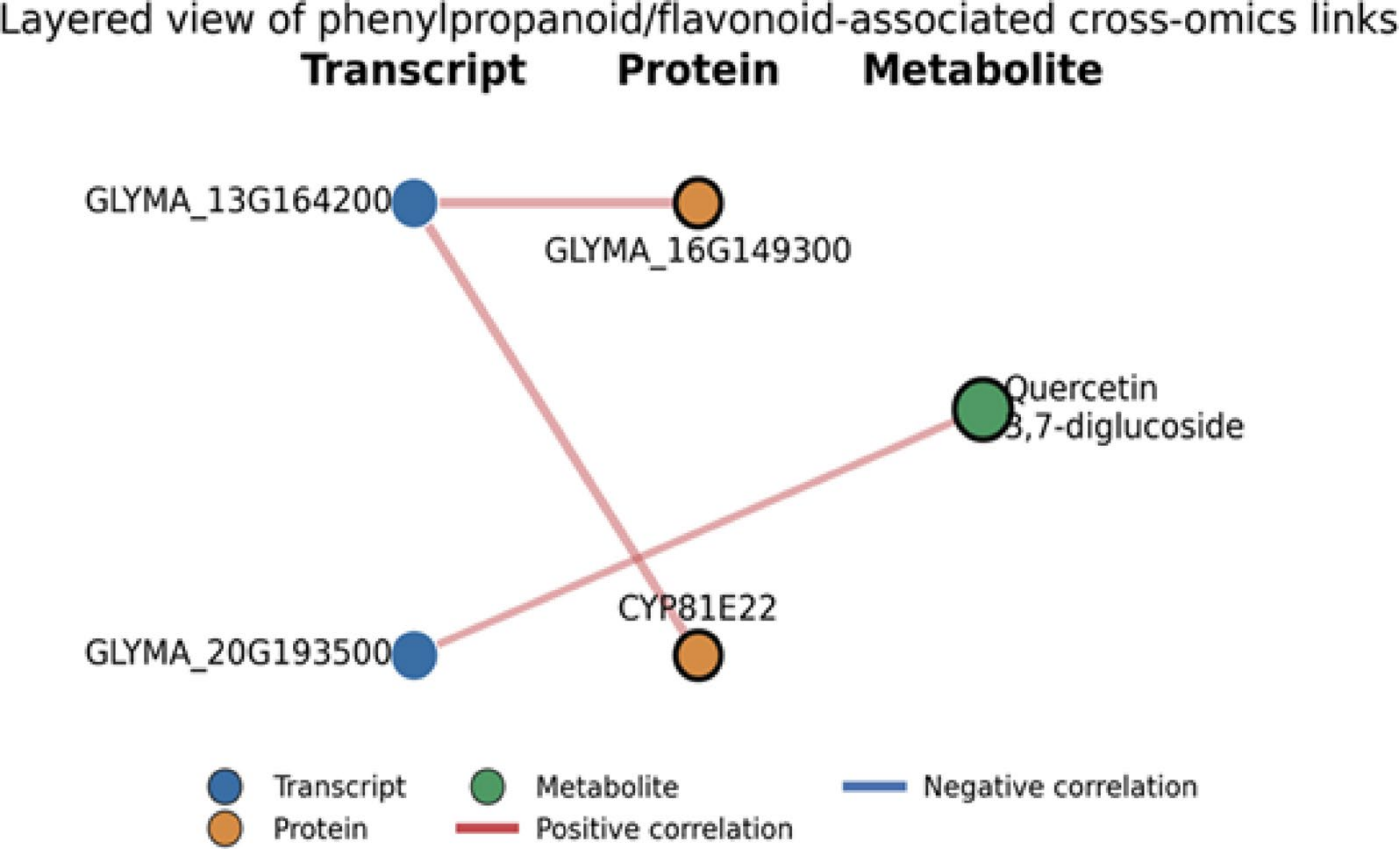
Cross-omics molecular interaction network centered on phenylpropanoid/flavonoid-associated features. Blue, orange and green nodes indicate transcripts, proteins and metabolites, respectively; red and blue edges indicate positive and negative Spearman correlations across matched CK and R-QD-50 groups

To move beyond simple pathway overlap, a cross-omics molecular interaction network was further constructed to evaluate sample-level associations among DEGs, DEPs and DAMs. The integrated network contained 889 connected nodes and 900 retained cross-omics associations, including 329 transcript-protein, 137 transcript-metabolite and 434 protein-metabolite links. Within the manuscript-relevant pathway subset, 65 connected focus nodes and 122 focus-associated edges were retained. The phenylpropanoid/flavonoid-centered subnetwork connected transcriptional regulators and pathway-related proteins, including chalcone synthase/isomerase family proteins, CYP81E-related isoflavonoid proteins, CYP73A11/trans-cinnamate 4-monooxygenase, peroxidases and caffeate O-methyltransferase-related proteins, with downstream secondary metabolites such as tetrahydroxyflavone, luteolinidin, quercetin 3,7-diglucoside, maackiain glucoside/malonate, corylifol A, 7-hydroxyisoflavone, glycitin, caffeic acid derivatives and p-coumaric acid-related compounds (Fig. 7). These cross-omics associations provide network-level evidence that ZnO QDs treatment remodels the secondary metabolic regulation rather than merely altering isolated pathway membership.

Phenylpropanoid biosynthesis displayed a complete transcriptome-proteome-metabolome regulatory chain. Key genes *PAL* and *C4H* were significantly upregulated in the transcriptome (|log_2_FC|> 1.5, *P* < 0.05). Corresponding synthase abundance increased by ~ 35% at the proteome level. Downstream metabolites including isoflavones, lignin, and phenolic acids increased by ~ 52% in the metabolome. These secondary metabolites are core functional food components of soybean, and their accumulation can significantly improve the development potential of soybean as a functional food raw material.

Among the three common pathways, phenylpropanoid biosynthesis acts as the key functional pathway for soybean quality improvement. Taurine and hypotaurine metabolism may contribute to antioxidant homeostasis, whereas butanoate metabolism shows limited association with core quality regulation.

qRT-PCR was carried out to validate the transcriptional changes of two hub genes (*PAL* and *C4H*) in phenylpropanoid biosynthesis screened from integrated multi-omics data. As shown in Fig. S5, the relative expression of *PAL* and *C4H* in R-QD-50 treated soybean seeds reaches 1.93-fold and 2.25-fold of that of the CK group (normalized to 1.00). Independent sample t-test confirms that both genes were extremely significantly upregulated under ZnO NMs (*** *P* < 0.001). The consistent upregulation trend of *PAL* and *C4H* at transcriptional level verifies the multi-omics finding that phenylpropanoid biosynthesis forms a complete positive regulatory cascade to improve soybean nutritional quality.

## Limitations and future perspectives

It is important to note that the conclusions of this study are derived from pot experiments under controlled environmental conditions, which presents certain limitations for direct extrapolation to the field production. Compared to field soils, potting soils are more homogeneous and have restricted root growth spaces, and they lack the leaching effects of natural rainfall. These factors may influence the migration, transformation, and bioavailability of ZnO NMs in the soil, thereby altering their actual impacts on soybean growth and quality. Furthermore, field production is subject to complex variables such as climatic fluctuations, pest and disease pressures, planting densities, and agricultural management practices. Therefore, the application protocol proposed in this study requires further validation and optimization through multi-year and multi-site field trials.

Additionally, this study did not establish a conventional zinc fertilizer control group, given that the core research aim was to elucidate the biological effects and molecular regulatory mechanisms of ZnO NMs on soybean quality, rather than to perform a head-to-head efficacy comparison between nano-scale and conventional zinc fertilizers. Accordingly, the application advantages of ZnO NMs discussed in this manuscript are primarily inferred from their unique nanoscale physicochemical properties and findings from existing literature, and the present work cannot directly demonstrate the superiority of ZnO NMs over conventional zinc fertilizers. Systematic comparative evaluation with conventional zinc fertilizers remains an important research direction in this field, to further clarify the practical application potential of ZnO NMs in agricultural production.

From the perspective of long-term agricultural application, ZnO NMs present both potential benefits and possible environmental risks. On the positive side, nano-zinc fertilizers exhibit higher nutrient use efficiency than conventional zinc fertilizers, which not only reduces the total zinc application rate, but also mitigates the risk of excessive zinc accumulation in soils and potential heavy metal contamination. Conversely, the long-term, large-scale application of ZnO NMs may affect soil microbial community structures, enzyme activities, and nutrient cycling processes. Their ecological risks to soil fauna and below-ground ecosystems remain to be systematically evaluated. Additionally, zinc accumulated in crop grains may enter the food chain, necessitating long-term tracking and assessment of its potential impacts on human health. Although the application concentrations used in this study fall within the safety ranges reported in existing literature, long-term positioning experiments are still required before large-scale promotion to systematically evaluate the ecological safety and environmental benefits of ZnO NMs.

## Conclusion

This study aimed to clarify the molecular mechanism by which ZnO NMs enhance soybean quality, and to determine the optimal application regime among NMs type, application mode and concentration. The results demonstrated that ZnO NMs regulate soybean growth and quality in a ZnO NMs type, application mode and concentration‑dependent manner. R‑QD‑50 was confirmed as the optimal treatment, which significantly improved root development, biomass accumulation, pod traits, seed yield and nutritional quality. Integrated multi‑omics analysis identified three common pathways across transcriptome, proteome and metabolome, among which phenylpropanoid biosynthesis was the only pathway forming a complete regulatory chain. This chain was characterized by significantly upregulated expression of *PAL* and *C4H*, increased abundance of corresponding synthases by approximately 35%, and enhanced accumulation of downstream metabolites including isoflavones, lignin and phenolic acids by ~ 52%. This pathway represents the core molecular mechanism underlying ZnO NMs‑improved soybean quality. By integrating multi-omics with conventional phenotypic and physiological analyses, this study establishes a systematic regulatory network for ZnO NMs-mediated soybean quality improvement, providing theoretical insights and a reference application regime for high-quality soybean production under controlled conditions. Overall, this study strengthens the theoretical basis for nano‑enabled crop improvement and lays a foundation for further field validation and practical optimization of ZnO NMs application in legume production.

## Supplementary Information

Below is the link to the electronic supplementary material.

Supplementary Material 1

## Data Availability

The datasets supporting the conclusions of this article are included within the article and its additional files. The raw transcriptome sequencing data generated in this study will be deposited in the NCBI Sequence Read Archive (SRA) database, and the raw mass spectrometry data of proteomics and metabolomics will be submitted to the MetaboLights database. The accession numbers will be provided and updated before the formal acceptance of the manuscript. All processed data supporting the findings of this study are included in the main text and Supplementary materials. The raw data are available from the corresponding author upon reasonable request.

## Abbreviations

ZnO NMs: Zinc oxide nanomaterials
ZnO NPs: ZnO nanoparticles
ZnO QDs: ZnO quantum dots
R-QD: Root application of ZnO QDs
R-NP: Root application of ZnO NPs
F-QD: Foliar application of ZnO QDs
F-NP: Foliar application of ZnO NPs
Se NPs: Selenium nanoparticles
TiO_2_ NPs: Titanium dioxide nanoparticles
EG: Ethylene glycol
CK: Blank control group
BH: Benjamini-Hochberg
QC: Quality control
CI: Confidence intervals
FWER: Family-wise error rate
PCA: Principal component analysis
SE: Standard error
KMO: The Kaiser–Meyer–Olkin
GO: Gene Ontology
DEGs: Differentially expressed genes
BP: Biological process
CC: Cellular component
MF: Molecular function
KEGG: Kyoto encyclopedia of genes and genomes
PLS-DA: Partial least squares discriminant analysis
DAMs: Differentially abundant metabolites
DEPs: Differentially expressed proteins
PAL: Phenylalanine ammonia lyase
C4H: Cinnamic acid-4-hydroxylase
DIA: Data-independent acquisition
CSAD: Cysteine Sulfinic Acid Decarboxylase
