# Supplementary material for: Integrating multi-omics with regular analyses to elucidate the effects of ZnO nanomaterials on improving soybean quality
Source: Bioresour Bioprocess. 2026 Aug 5;13(1):109. doi: 10.1186/s40643-026-01109-1 (PMC13442772; doi:10.1186/s40643-026-01109-1)
Supplement: Supplementary file 1 [file 40643_2026_1109_MOESM1_ESM.docx]

**Supplementary Information**

**Integrating multi-omics with regular analyses to elucidate the effects of ZnO nanomaterials on improving soybean quality**

Xinqi Hu, Yi Zhao, Shixin Cai, Yuting Zhang, Xiaoli Wang, Nandi Zhou*

School of Biotechnology and Key Laboratory of Carbohydrate Chemistry and Biotechnology of Ministry of Education, Jiangnan University, Wuxi 214122, China

*Corresponding author. E-mail: zhounandi@jiangnan.edu.cn

**Experimental section**

**Transcriptomics analysis**

***RNA extraction, quantification, library preparation and sequencing***

The total RNA of soybean seeds was extracted using TransZol UP (TransGen Biotech, Beijing, China). RNA integrity was assessed by 1.5% agarose gel electrophoresis, and RNA purity was determined using a NanoDrop spectrophotometer. Subsequently, the construction and quality inspection of the transcriptome library were performed according to the instructions of VAHTS Universal V8 RNA-seq Library Prep Kit for Illuminubit. Briefly, mRNA with poly (A) tail was enriched using oligo (dT) magnetic beads, and mRNA was randomly fragmented by divalent cations in fragmentation buffer at 94℃ for 5 min. First-strand cDNA was synthesized in an M-MuLV reverse transcriptase system using fragmented mRNA as the template and random oligonucleotides as the primers. Then the RNA template was degraded by RNase H, and second-strand cDNA was synthesized from deoxyribonucleoside triphosphates (dNTPs) in DNA polymerase I system. After purification, the double-stranded cDNA was subjected to terminal repair, adenylation (A-tailing), and ligation of sequencing adapters. cDNA fragments of 370~420 bp were selected using AMPure XP beads. PCR amplification was performed with specific primers under the following conditions: 15 cycles of 98℃ for 10 s, 60℃ for 30 s, and 72℃ for 30 s. The PCR products were purified again using AMPure XP beads. After the library was constructed, Qubit 4.0 Fluorometer (Thermo Fisher Scientific, USA) was used for preliminary quantification. The library was diluted to 1.5 ng/μL, and then the insert size of the library was detected using Agilent 2100 Bioanalyzer (Agilent Technologies, USA). After the insert size met the expectation, the effective concentration of the library was accurately quantified by qRT-PCR (effective concentration ≥ 2 nM). Qualified libraries were equimolarly pooled based on their effective concentrations to achieve the target sequencing data volume followed by sequencing on an Illumina NovaSeq 6000 sequencing platform. Base calling was performed using Casava software (Illumina, USA), generating 150 bp paired-end reads.

***Data quality control, sequence alignment, and gene expression quantification***

Firstly, quality control of raw data was performed using Fastp software (v0.23.2, https://github.com/OpenGene/fastp). This process mainly included the removal of adapter-contaminated reads, reads containing N (N indicates undetermined base information), and low-quality reads (Phred quality score < 20). Meanwhile, Q20 scores, Q30 scores, and GC content of the clean data were calculated to evaluate data quality. All subsequent analyses were performed using high-quality clean data. The reference genome index was constructed using HISAT2 (v2.2.1, https://doi.org/10.1038/s41587-019-0201-4), and clean reads were aligned to the Glycine max reference genome (v2.1, <https://ensembl.gramene.org/Glycine_max/Info/Index>) (Sirén et al., 2014). The number of reads mapped to each gene was calculated using FeatureCounts (v2.0.0, https://github.com/topics/featurecounts), followed by calculation of FPKM (fragments per kilobase of transcript per million mapped fragments) values for each gene using StringTie (v2.0.0, https://ccb.jhu.edu/software/stringtie/index.shtml) based on gene length and the number of mapped reads, which was used to quantify gene expression levels (Kovaka et al., 2019; Pertea et al., 2016; Shinder et al., 2025; Shumate et al., 2022).

***Gene expression differences and enrichment analysis***

Differential expression analysis between ZnO NMs-treated and control soybean seeds was conducted using DESeq2 software (v1.20.0) (Muzellec et al., 2023). The Benjamini-Hochberg method was used to adjust the obtained P-values to control the false discovery rate (FDR). FDR < 0.05 and |log₂FC| > 1 were set as the screening criteria for DEGs using EdgeR (https://bioconductor.org/packages/release/bioc/html/edgeR.html). Gene Ontology (GO) and Kyoto Encyclopedia of Genes and Genomes (KEGG) pathway enrichment analyses were performed using ClusterProfiler software (v4.0.2, https://bioconductor.org/packages/release/bioc/html/clusterProfiler.html) with Fisher’s exact test. To control the false positive rate, multiple testing correction was performed using the BH (FDR) method. A corrected P value threshold of 0.05 was used to define significantly enriched GO terms and KEGG pathways in DEGs. Gene Set Enrichment Analysis (GSEA) bypassedthe requirement for explicit DEG thresholds by leveraging pre-defined gene sets (derived from functional annotations or prior experimental data). Genes were ranked by their differential expression between the two sample groups, and the enrichment of target gene sets at the extremities of this ranked list was evaluated. By prioritizing coordinated expression changes of gene sets over individual genes, GSEA captureed subtle transcriptional variations, facilitating more reliable biological interpretations. GSEA was performed on soybean GO and KEGG datasets using GSEA software (v4.3.2, <http://www.broadinstitute.org/gsea/index.jsp>) (Mootha et al., 2003).

**Proteomics analysis**

***Protein extraction, quantification, SDS-PAGE, proteolysis and desalination***

The soybean samples were thawed on ice. An appropriate amount of the thawed soybean sample was transferred into a 2 mL centrifuge tube, and then urea lysis buffer and protease inhibitor were added for protein extraction. The centrifuge tube containing the sample was vortexed for 3 min using Vortex mixer (Haimen Kylin-bell Lab Instruments Co., Ltd., China), placed on ice and disrupted using an ultrasonic cell disruptor at a vibration frequency of 30 Hz, with a cycle of 10 s sonication and 10 s interval, repeated 6 times. The samples were heated at 95℃ for 5 min to denature proteins and then centrifuged at 4℃, 12,000 rpm for 20 min. The supernatant was collected for subsequent experiments. The BCA method was used to determine the concentration of the aforementioned extracted proteins. Based on the quantitative results, an appropriate amount of sample was mixed with SDS-PAGE loading buffer for SDS-PAGE analysis. The protein bands and their distribution were visualized by staining with Coomassie Brilliant Blue R-250. An appropriate amount of protein was taken for enzymatic digestion, and urea lysis buffer was added to ensure a total volume of 50 μL. Then, 6 μL of reduction and alkylation reagentwas added, and the mixture was vortexed for 30 s, centrifuged for 10 s, and incubated at 95 ℃ for 5 min. Trypsin was then added at a trypsin-to-protein mass ratio of 1:50, followed by vortexing for 30 s, centrifuging for 10 s, and incubating at 37 ℃ for 12 h. At the end of incubation, digestion termination reagent (10% trifluoroacetic acid, TFA) was added, followed by desalination. The filtrate was collected and vacuum freeze-dried to obtain the peptides.

***Peptide analysis by liquid chromatography-mass spectrometry (LC-MS)***

Based on the peptide quantification results, the peptides were solubilized with mass spectrometry loading buffer (0.1% formic acid) for LC-MS analysis. Data acquisition was performed using Xcalibur software (Thermo, USA). The LC analysis was carried out on a Vanquish Neo system (Thermo, USA) equipped with a homemade C18 column (15 cm×100 μm, 1.7 μm). Mass spectrometric detection was conducted on an Astral mass spectrometer (Thermo, USA). The flow rate was set at 300 nL/min, and the column temperature was maintained at 40 ℃. Mobile phase A: 98% water, 2% acetonitrile, and 0.1% formic acid (v/v); mobile phase B: 80% acetonitrile, 20% water, and 0.1% formic acid (v/v). The gradient elution program was as follows: 0~1 min, 8%~17% mobile phase B; 1~5.5 min, 17%~55% mobile phase B; 5.5~7 min, 55%~99% mobile phase B; 7~8 min, 99% mobile phase B.

***Proteome data analysis***

Significantly DEPs were identified based on P < 0.05 and |log₂FC| > 1.5. Functional annotation and enrichment analysis of the screened DEPs were performed using the GO and KEGG databases to evaluate the enrichment significance level in specific GO terms or KEGG pathways. In addition, this study also used the InterPro database for domain annotation and enrichment analysis, WoLF PSORT software (https://wolfpsort.hgc.jp/) for subcellular localization prediction, and the PlantTFDB 5.0 database for transcription factor prediction of the target proteins. In addition, Protein-Protein Interaction (PPI) was used to found the direct or indirect interaction network between the target protein IDs in the String database (https://www.string-db.org/). Since traditional enrichment analysis focuses on comparing gene expression differences between two groups and concentrates on a few significantly upregulated or downregulated genes, it has certain limitations. Therefore, this study also employed GSEA. GSEA-GO and GSEA-KEGG analyses were performed using GSEA software (http://software.broadinstitute.org/gsea/index.jsp) on the gene names corresponding to all identified proteins (Subramanian et al., 2005).

**Metabolomics analysis**

***Metabolite extraction***

30 mg fresh soybean sample was weighed and put into a 2 mL centrifuge tube, and 600 µL methanol (Fisher Scientific, Loughborough, UK) containing 4 ppm 2-chloro-L-phenylalanine (Aladdin, Shanghai, China) was added. The mixture was vortexed for 30 s, followed by the addition of steel balls. The tube was then placed into a tissue grinder (Zhejiang Meibi Experiment Equipment Co., Ltd., China) and ground at 50 Hz for 120 s. After ultrasonic extraction at room temperature for 10 min, the homogenate was centrifuged at 4 °C, 12000 rpm for 10 min. The supernatant was filtered through 0.22 μm microporous membrane filters (Tianjin Jinteng Experiment Equipment Co., Ltd, China), and the filtered solution was added to the detection bottle for LC-MS detection (Turroni et al., 2016).

***LC-MS analysis of metabolic extracts***

The LC analysis was performed on a Vanquish UHPLC System (Thermo Fisher Scientific, USA). Chromatography was carried out with an ACQUITY UPLC ^®^ HSS T3 (2.1×100 mm, 1.8 µm) (Waters, Milford, MA, USA). The column maintained at 40 ℃. The flow rate and injection volume were set at 0.3 mL/min and 2 μL, respectively. For LC-ESI (+)-MS analysis, mobile phases were (A) 0.1% formic acid in water and (B) 0.1% formic acid in acetonitrile (v/v). The separation was performed using the following gradient program: 0–1 min, 10% B; 1–5 min, 10%–98% B; 5–6.5 min, 98% B; 6.5–6.6 min, 98%–10% B; 6.6–8 min, 10% B； For LC‑ESI (−)‑MS analysis, mobile phases were (A) 5 mM ammonium formate in water and (B) acetonitrile. The separation was performed using the following gradient program: 0–1 min, 10% B; 1–5 min, 10%–98% B; 5–6.5 min, 98% B; 6.5–6.6 min, 98%–10% B; 6.6–8 min, 10% B (Zelena et al., 2009). Mass spectrometric detection of metabolites was performed on Orbitrap Exploris 120 (Thermo Fisher Scientific, USA) with ESI ion source. Simultaneous MS1 and MS/MS (Full MS-ddMS2 mode, data-dependent MS/MS) acquisition was used. The parameters were as follows: sheath gas pressure, 40 arbitrary units (arb); auxiliary gas flow, 10 arb; spray voltage, 3.50 kV and -2.50 kV for ESI(+) and ESI(-), respectively; capillary temperature, 325 °C; MS1 range, m/z 100-1000; MS1 resolving power, 60000 FWHM; number of data-dependent scans per cycle, 4; MS/MS resolving power, 15000 FWHM; normalized collision energy, 30%; dynamic exclusion time, automatic (Zelena et al., 2009).

***Metabolome data analysis***

Raw metabolomics data were converted to mzXML format using MSConvert (ProteoWizard v3.0.8789) and processed with the R package XCMS (v3.12.0) for feature detection (parameters: ppm=15, peakwidth=c (5, 30), mzdiff=0.01, method=centWave), retention time correction, and alignment (Navarro-Reig et al., 2015; Rasmussen et al., 2022; Smith et al., 2006). Batch effects were mitigated by QC sample-based normalization (prepared by pooling equal volumes of all extracted samples to correct for systematic errors from the LC-MS system and batch-to-batch variations), and metabolites with relative standard deviation (RSD) > 30% in QC samples were excluded. Metabolite identification was accomplished by matching accurate mass and MS/MS data against public databases (MassBank (http://www.massbank.jp/), KEGG (https://www.genome.jp/kegg/), LipidMaps (http://www.lipidmaps.org), mzCloud (https://www.mzcloud.org)) and a custom database from Tgene Biotech (Shanghai, China).(Abdelrazig et al., 2020; Horai et al., 2010; Kanehisa & Goto, 2000; Navarro-Reig et al., 2015; Sud et al., 2007) The molecular weight of each metabolite was determined from the m/z value of the parent ion, and the molecular formula was predicted using ppm tolerance and adduct ion information prior to database matching. MS/MS data were further validated by comparing fragment ions with corresponding entries in the databases to confirm structural annotations. Unsupervised PCA and supervised PLS-DA/OPLS-DA were conducted using the R package ropls (v1.22.0) for group discrimination; differential metabolites were screened based on *P* < 0.05 and VIP (OPLS-DA Variable Importance in the Projection) > 1 (Thévenot et al., 2015). Pathway enrichment analysis was implemented *via* hypergeometric distribution and topological analysis, with identified metabolites mapped to KEGG pathways and visualized using KEGG Mapper for functional interpretation (Xia & Wishart, 2011).

**Supplementary multi-omics network analysis**

**Input data and feature selection**

Cross-omics association analysis was performed using matched quantitative transcriptomic, proteomic and non-targeted metabolomic profiles from 12 soybean seed samples (six CK and six R-QD-50 samples). Candidate features were defined as the union of differential features and pathway-focused features. Differential transcripts were taken from the original DEG table. Differential proteins for network candidate screening included proteins labelled UP or DOWN in the original differential table, as well as proteins with *P* < 0.05 and |log_2_FC| ≥ log_2_(1.2). This relaxed threshold was applied exclusively for network construction to retain more pathway-relevant proteins with moderate expression changes, and did not alter the official DEP identification criteria (|log_2_FC| > 1.5, FDR < 0.05) reported in the main text. Significant differential metabolites were taken from the original metabolite annotation table.

Features annotated to phenylpropanoid biosynthesis, flavonoid biosynthesis, isoflavonoid biosynthesis, butanoate metabolism or taurine and hypotaurine metabolism, together with features whose annotations matched phenylpropanoid, flavonoid, isoflavonoid, *PAL* or *C4H*-related keywords, were added to preserve full coverage of the core pathways discussed in the manuscript. This pathway-focused expansion ensured comprehensive capture of the regulatory cascade even for features not meeting strict differential expression thresholds. The resulting candidate set comprised 1,050 transcripts, 1,269 proteins and 476 metabolites.

**Data preprocessing**

For each feature, missing quantitative values were imputed with one-half of the smallest positive observed value for that feature. If a feature contained no positive value but had observed values, the smallest observed value was used. Features with no observed value across all samples were set to 0. All feature profiles were then transformed as log_2_(x + 1). Features with a standard deviation ≤ 1 ×10^-12^ across the 12 samples were removed before correlation analysis. This imputation strategy is widely adopted in multi-omics correlation studies to minimize bias from missing observations while retaining pathway-relevant features.

**Cross-omics correlation and edge filtering**

Only inter-layer feature pairs were tested, including transcript-protein, transcript-metabolite and protein-metabolite pairs. Within-layer pairs were not included in the analysis. Spearman rank correlation coefficients were calculated across the 12 matched samples. Two-sided *P* values were derived from the t-distribution approximation for Spearman rho, with degrees of freedom df = n － 2 = 10.

A hierarchical threshold screening strategy was applied from strict to relaxed criteria. A relaxed candidate edge required |rho| ≥ 0.70 and *P* ≤ 0.05. The final filter was evaluated in the following order: |rho| ≥ 0.90 and *P* ≤ 0.002; |rho| ≥ 0.85 and *P* ≤ 0.005; |rho| ≥ 0.80 and *P* ≤ 0.010; |rho| ≥ 0.75 and *P* ≤ 0.020; or |rho| ≥ 0.70 and *P* ≤ 0.050. The first rule yielding at least 80 valid edges was selected. The 80-edge minimum was predefined to ensure a sufficiently dense network for functional interpretation while maintaining high stringency. In the present analysis, the strictest rule (|rho| ≥ 0.90, two-sided *P* ≤ 0.002) was selected.

Given the exploratory nature of the association network and the relatively small sample size, raw *P* values were used for edge filtering in combination with a very strict correlation coefficient threshold. This joint threshold strategy effectively controls false positive rates without excessive loss of biologically relevant cross-omics associations that would be caused by strict multiple testing correction.

Among 63,785 relaxed candidate edges, final edges were ranked by descending |rho| and ascending P value, and capped at 900 edges. This upper limit was set to balance network coverage and interpretability, and to keep the exported network tractable for downstream analysis.

**Node, edge and visualization definitions**

Each node represented one independent transcript, protein or metabolite. Node tables record the feature identifier, annotation, omics class, differential information, pathway tag, focus-node flag, degree and weighted degree. An edge represented a retained inter-omics Spearman association across the matched samples, rather than a validated physical interaction or causal regulatory relationship. Edges with rho ≥ 0 were labelled positive and those with rho < 0 were labelled negative. Degree was the number of retained edges incident on a node, and weighted degree was the sum of |rho| over its retained edges.Focus nodes were identified by the pathway and keyword rules above for pathway-centred ranking and visualization only. Focus status was not an additional statistical significance criterion. The layered subnetwork displayed in **Fig. 7** of the main text is a simplified visualization of the focus node set, showing the strongest links ranked by |rho| and *P* value.

**Software**

The analysis was implemented in a Python (v3.10) environment using Pandas (v2.3.3), NumPy (v2.2.6), SciPy (v1.15.3), NetworkX (v3.4.2), Matplotlib (v3.10.9) and Openpyxl (v3.1.5). The network summarizes sample-level statistical associations and should not be interpreted as evidence of direct molecular binding or causality.

**Fig. S1** Characterization of ZnO QDs. (**a**) TEM, (**b**) Particle size distribution histogram, (**c**) Fluorescence spectrum and UV visible spectrum, (**d**) XRD, (**e**) XPS and (**f**) FTIR images of ZnO QDs.

**Fig. S2** Material characterization of ZnO NPs. (**a**)TEM, (**b**) Particle size distribution histogram, (**c**) UV visible spectrum**,** (**d**) XRD, (**e**) XPS and (**f**) FTIR images of ZnO NPs.

**Fig. S3** Schematic diagram of the nitrogen metabolism pathway.

**Fig. S4** Schematic diagram of the arginine biosynthesis pathway.

**Fig. S5** Relative expression of core phenylpropanoid genes *PAL* and *C4H.* Data represent mean ± SD, n = 3. Independent sample t-test, *** *P* < 0.001.

**Table S1** qRT-PCR information and primer sequences.

| Genes | Sequences |
| --- | --- |
| *PAL*-F | GGAAGCAACTACCATCACCA |
| *PAL*-R | CTCCCCAGTTTAACGGATCACT |
| *C4H*-F | AACCCAACCAAACTGAACCA |
| *C4H*-R | AGGGTCTTTTCCAGAAGGAGG |
| TUBB3-FP (Reference Gene)  TUBB3-RP (Reference Gene) | GCGGCACCTATCTGTTTGTT ACACCTTGACCTCAACACAGT |

**Table S2** Three omics combined pathway.

| KEGG ID (Transcriptome-proteome joint analysis) | KEGG ID（Metabolomics) | KEGG ID (Intersection) |
| --- | --- | --- |
| Starch and sucrose metabolism | C5-Branched dibasic acid metabolism | Taurine and hypotaurine metabolism |
| Phenylpropanoid biosynthesis | One carbon pool by folate | Butanoate metabolism |
| Photosynthesis - antenna proteins | Efferocytosis | Phenylpropanoid biosynthesis |
| Oxidative phosphorylation | Nitrogen metabolism |  |
| Photosynthesis | Arginine biosynthesis |  |
| Protein processing in endoplasmic reticulum | Taurine and hypotaurine metabolism |  |
| Butanoate metabolism | Linoleic acid metabolism |  |
| Biosynthesis of various plant secondary metabolites | Alanine, aspartate and glutamate metabolism |  |
| Cysteine and methionine metabolism | Glycolysis / Gluconeogenesis |  |
| Taurine and hypotaurine metabolism | Pyruvate metabolism |  |
|  | Sphingolipid metabolism |  |
|  | Glutathione metabolism |  |
|  | Porphyrin metabolism |  |
|  | Propanoate metabolism |  |
|  | Lipoic acid metabolism |  |
|  | Galactose metabolism |  |
|  | Histidine metabolism |  |
|  | Butanoate metabolism |  |
|  | Glycine, serine and threonine metabolism |  |
|  | Phenylalanine metabolism |  |
|  | Flavone and flavonol biosynthesis |  |
|  | Aminoacyl-tRNA biosynthesis |  |
|  | Fructose and mannose metabolism |  |
|  | Nicotinate and nicotinamide metabolism |  |
|  | Ascorbate and aldarate metabolism |  |
|  | Phenylpropanoid biosynthesis |  |
|  | Carotenoid biosynthesis |  |
|  | Pyrimidine metabolism |  |
|  | Glyoxylate and dicarboxylate metabolism |  |
|  | Arginine and proline metabolism |  |
|  | D-Amino acid metabolism |  |
|  | ABC transporters |  |
|  | Glucosinolate biosynthesis |  |
|  | Tryptophan metabolism |  |

**References**

Abdelrazig, S., Safo, L., Rance, G. A., Fay, M. W., Theodosiou, E., Topham, P. D., Kim, D. H., & Fernández-Castané, A. 2020. Metabolic characterisation of Magnetospirillum gryphiswaldense MSR-1 using LC-MS-based metabolite profiling. *RSC Advances*, *10*(54), 32548-32560. https://doi.org/10.1039/d0ra05326k.

Horai, H., Arita, M., Kanaya, S., Nihei, Y., Ikeda, T., Suwa, K., Ojima, Y., Tanaka, K., Tanaka, S., Aoshima, K., Oda, Y., Kakazu, Y., Kusano, M., Tohge, T., Matsuda, F., Sawada, Y., Hirai, M. Y., Nakanishi, H., Ikeda, K., Akimoto, N., Maoka, T., Takahashi, H., Ara, T., Sakurai, N., Suzuki, H., Shibata, D., Neumann, S., Iida, T., Tanaka, K., Funatsu, K., Matsuura, F., Soga, T., Taguchi, R., Saito, K., & Nishioka, T. 2010. MassBank: a public repository for sharing mass spectral data for life sciences. *Journal of Mass Spectrometry*, *45*(7), 703-714. https://doi.org/10.1002/jms.1777.

Kanehisa, M., & Goto, S. 2000. KEGG: kyoto encyclopedia of genes and genomes. *Nucleic Acids Research*, *28*(1), 27-30. https://doi.org/10.1093/nar/28.1.27.

Kovaka, S., Zimin, A. V., Pertea, G. M., Razaghi, R., Salzberg, S. L., & Pertea, M. 2019. Transcriptome assembly from long-read RNA-seq alignments with StringTie2. *Genome Biology*, *20*(1), 278. https://doi.org/10.1186/s13059-019-1910-1.

Mootha, V. K., Lindgren, C. M., Eriksson, K. F., Subramanian, A., Sihag, S., Lehar, J., Puigserver, P., Carlsson, E., Ridderstråle, M., Laurila, E., Houstis, N., Daly, M. J., Patterson, N., Mesirov, J. P., Golub, T. R., Tamayo, P., Spiegelman, B., Lander, E. S., Hirschhorn, J. N., Altshuler, D., & Groop, L. C. 2003. PGC-1alpha-responsive genes involved in oxidative phosphorylation are coordinately downregulated in human diabetes. *Nature Genetics*, *34*(3), 267-273. https://doi.org/10.1038/ng1180.

Muzellec, B., Teleńczuk, M., Cabeli, V., & Andreux, M. 2023. PyDESeq2: a python package for bulk RNA-seq differential expression analysis. *Bioinformatics*, *39*(9). https://doi.org/10.1093/bioinformatics/btad547.

Navarro-Reig, M., Jaumot, J., García-Reiriz, A., & Tauler, R. 2015. Evaluation of changes induced in rice metabolome by Cd and Cu exposure using LC-MS with XCMS and MCR-ALS data analysis strategies. *Analytical and Bioanalytical Chemistry*, *407*(29), 8835-8847. https://doi.org/10.1007/s00216-015-9042-2.

Pertea, M., Kim, D., Pertea, G. M., Leek, J. T., & Salzberg, S. L. 2016. Transcript-level expression analysis of RNA-seq experiments with HISAT, StringTie and Ballgown. *Nature Protocols*, *11*(9), 1650-1667. https://doi.org/10.1038/nprot.2016.095.

Rasmussen, J. A., Villumsen, K. R., Ernst, M., Hansen, M., Forberg, T., Gopalakrishnan, S., Gilbert, M. T. P., Bojesen, A. M., Kristiansen, K., & Limborg, M. T. 2022. A multi-omics approach unravels metagenomic and metabolic alterations of a probiotic and synbiotic additive in rainbow trout (Oncorhynchus mykiss). *Microbiome*, *10*(1), 21. https://doi.org/10.1186/s40168-021-01221-8.

Shinder, I., Pertea, G., Hu, R., Rudnick, Z., & Pertea, M. 2025. StringTie3 improves total RNA-seq assembly by resolving nascent and mature transcripts. *bioRxiv*. https://doi.org/10.1101/2025.05.21.655404.

Shumate, A., Wong, B., Pertea, G., & Pertea, M. 2022. Improved transcriptome assembly using a hybrid of long and short reads with StringTie. *PLOS Computational Biology*, *18*(6), e1009730. https://doi.org/10.1371/journal.pcbi.1009730.

Sirén, J., Välimäki, N., & Mäkinen, V. 2014. Indexing graphs for path queries with applications in genome research. *IEEE/ACM Transactions on Computational Biology and Bioinformatics*, *11*(2), 375-388. https://doi.org/10.1109/tcbb.2013.2297101.

Smith, C. A., Want, E. J., O'Maille, G., Abagyan, R., & Siuzdak, G. 2006. XCMS: processing mass spectrometry data for metabolite profiling using nonlinear peak alignment, matching, and identification. *Analytical Chemistry*, *78*(3), 779-787. https://doi.org/10.1021/ac051437y.

Subramanian, A., Tamayo, P., Mootha, V. K., Mukherjee, S., Ebert, B. L., Gillette, M. A., Paulovich, A., Pomeroy, S. L., Golub, T. R., Lander, E. S., & Mesirov, J. P. 2005. Gene set enrichment analysis: a knowledge-based approach for interpreting genome-wide expression profiles. *Proceedings of the National Academy of Sciences of the United States of America*, *102*(43), 15545-15550. https://doi.org/10.1073/pnas.0506580102.

Sud, M., Fahy, E., Cotter, D., Brown, A., Dennis, E. A., Glass, C. K., Merrill, A. H., Jr., Murphy, R. C., Raetz, C. R., Russell, D. W., & Subramaniam, S. 2007. LMSD: LIPID MAPS structure database. *Nucleic Acids Research*, *35*(Database issue), D527-532. https://doi.org/10.1093/nar/gkl838.

Thévenot, E. A., Roux, A., Xu, Y., Ezan, E., & Junot, C. 2015. Analysis of the human adult urinary metabolome variations with age, body mass index, and gender by implementing a comprehensive workflow for univariate and OPLS statistical analyses. *Journal of Proteome Research*, *14*(8), 3322-3335. https://doi.org/10.1021/acs.jproteome.5b00354.

Turroni, S., Fiori, J., Rampelli, S., Schnorr, S. L., Consolandi, C., Barone, M., Biagi, E., Fanelli, F., Mezzullo, M., Crittenden, A. N., Henry, A. G., Brigidi, P., & Candela, M. 2016. Fecal metabolome of the Hadza hunter-gatherers: a host-microbiome integrative view. *Scientific Reports*, *6*, 32826. https://doi.org/10.1038/srep32826.

Xia, J., & Wishart, D. S. 2011. Web-based inference of biological patterns, functions and pathways from metabolomic data using MetaboAnalyst. *Nature Protocols*, *6*(6), 743-760. https://doi.org/10.1038/nprot.2011.319.

Zelena, E., Dunn, W. B., Broadhurst, D., Francis-McIntyre, S., Carroll, K. M., Begley, P., O'Hagan, S., Knowles, J. D., Halsall, A., Wilson, I. D., & Kell, D. B. 2009. Development of a robust and repeatable UPLC-MS method for the long-term metabolomic study of human serum. *Analytical Chemistry*, *81*(4), 1357-1364. https://doi.org/10.1021/ac8019366.
